# Chemical Strategies for Multistimuli Responsive Dynamic Covalent Materials in Regenerative Medicine

**DOI:** 10.1002/adhm.71721

**Published:** 2026-09-17

**Authors:** Saurabh Joshi, Matthew B. Baker, Dominik Schauenburg

**Affiliations:** ^1^ Department of Instructive Biomaterials Engineering MERLN Institute for Technology‐Inspired Regenerative Medicine Maastricht University Maastricht The Netherlands

**Keywords:** adaptive hydrogels, dynamic covalent chemistry, extracellular matrix mimicry, multi‐stimuli responsive biomaterials, regenerative medicine, self‐healing materials

## Abstract

Regenerative medicine requires biomaterials that can function within complex and dynamically changing biological environments. Dynamic covalent chemistry provides a compelling foundation for such materials by combining covalent stability with reversible bond exchange, enabling adaptability, self‐healing, and stimulus‐responsive behavior. Dynamic covalent biomaterials have therefore emerged as promising platforms for tissue scaffolds, wound healing, and controlled delivery applications. Although many existing systems respond to a single stimulus, such as pH, redox conditions, or enzymatic activity, biological environments are inherently multiresponsive, integrating mechanical, chemical, and biochemical cues simultaneously. As a result, single‐stimulus dynamic materials often fail to achieve predictable performance in vivo. This review focuses on chemical strategies for engineering multistimuli responsive dynamic covalent materials that more closely reflect the complexity of native regenerative processes. We discuss key reversible covalent motifs, their thermodynamic and kinetic characteristics, and how these can be combined through orthogonal chemistries, multicomponent networks, and hierarchical architectures to achieve tunable mechanics, stress relaxation, degradation, and biofunctionality. Finally, we outline current challenges and opportunities, emphasizing the need for expanded biocompatible chemistries and rational design principles to advance multistimuli responsive dynamic covalent materials toward translational regenerative medicine.

## Graphical abstract

Multistimuli responsive dynamic covalent materials integrate reversible chemistry with the complex chemical, mechanical, and biochemical cues of regenerative environments. This review highlights chemical strategies based on orthogonal dynamic bonds, multicomponent networks, and hierarchical architectures to regulate mechanics, degradation, and biofunctionality, providing design principles for adaptive biomaterials with enhanced translational potential.

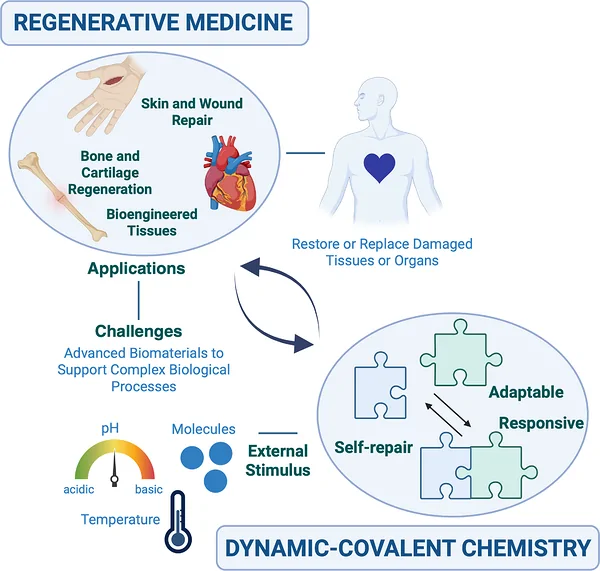

## 1 Introduction

Regenerative medicine represents a transformative field of biomedical science that seeks to restore or replace damaged tissues and organs, offering hope for treating conditions that currently lack effective therapies [1, 2, 3, 4]. By leveraging the body's natural repair mechanisms or providing engineered substitutes, it offers life‐changing solutions to diseases and injuries that currently have limited effective treatments. Applications range from repairing skin wounds and regenerating bone or cartilage to addressing organ failure through bioengineered tissues [5, 6, 7, 8]. The significance of regenerative medicine extends beyond healthcare, holding profound societal implications as populations age and the demand for novel treatments escalates. However, despite its promise, the field faces substantial challenges, many of which hinge on the development of advanced biomaterials capable of supporting complex environments [9, 10]. Meeting these challenges requires materials that not only perform passively but also actively engage with the biological milieu, promoting repair, integration, and function (Figure 1).

**FIGURE 1:**
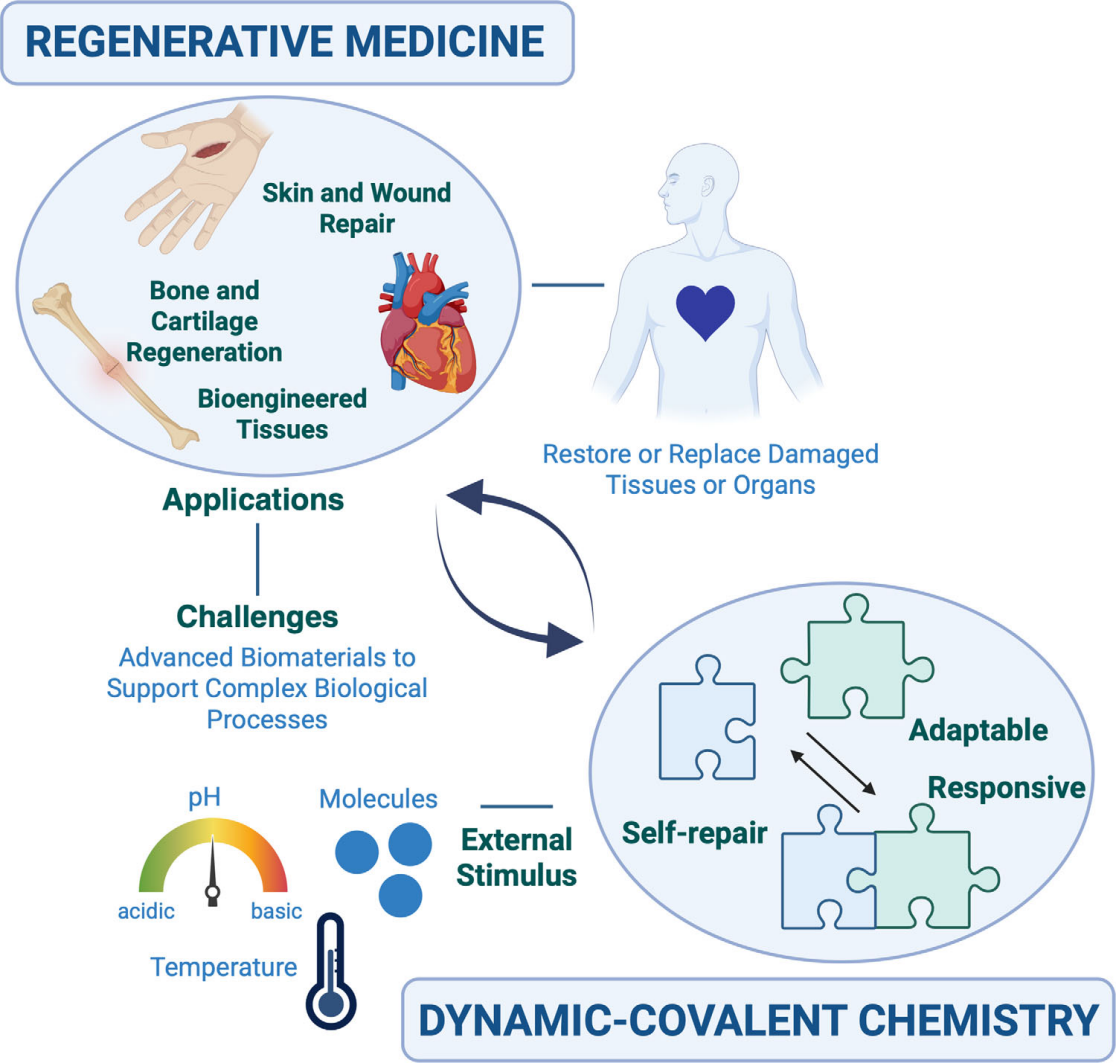
Synergy between regenerative medicine and its key challenges with dynamic covalent chemistry and its advantages. The schematic illustrates how dynamic covalent materials can address major obstacles in regenerative medicine by offering reversible bonding, tunable mechanical properties, and stimulus‐responsiveness.

Dynamic‐covalent chemistry (DCvC) has emerged as a transformative approach for designing advanced biomaterials, particularly for applications in regenerative medicine [11, 12]. At its core, DCvC relies on reversible covalent bonds that can form and break under thermodynamic equilibrium, enabling materials to dynamically reorganize their structures and properties in response to external stimuli such as changes in pH, temperature, or the presence of specific molecules [11, 13, 14, 15]. This unique combination of covalent bond strength and reversibility creates a framework for developing materials that are adaptable, responsive, and capable of self‐repair—qualities essential for addressing the complex demands of regenerative therapies.

Unlike traditional covalent bonds, which are static and permanent, dynamic‐covalent bonds offer flexibility and responsiveness, making them highly suited for biological systems where environmental conditions are constantly changing [11]. Examples of such bonds include imines (Schiff bases) [16, 17, 18], hydrazones [19, 20, 21], oximes [22, 23], boronic esters [24], and other boronic acid ligands [25, 26, 27], disulfide bonds [28, 29, 30], (thio)esters [31, 32, 33], Michael additions [34, 35], and reversible Diels–Alder adducts [36, 37], each with distinct reactivity profiles and sensitivities that allow for the customization of materials for specific applications (chapter 3). For instance, in tissue scaffolds, materials must withstand and adapt to mechanical stresses, biochemical signals, and cellular activities during regeneration [38, 39, 40]. Static materials often fail to meet these demands, whereas DCvC‐based materials can actively adjust their mechanical and structural properties in real time, maintaining their functionality and supporting tissue repair. Moreover, the self‐healing capabilities inherent to DCvC systems allow these materials to autonomously repair damage, extending their lifespan and reducing the need for repeated interventions [41, 42]. This feature is particularly valuable for applications in mechanically active environments, such as cartilage, joints, or cardiovascular tissues, where durability and adaptability are critical.

Due to adaptability and dynamic nature of DCvC, the field has gained significant attention for developing multiresponsive systems to mimic nature, as biological systems are multiresponsive [43, 44]. For example, proteins and enzymes exhibit responsiveness to pH, ionic concentration, and redox environment, cellular pathways are regulated by force‐dependent conformational changes, and degradation processes are controlled by multiple overlapping chemical cues [45, 46, 47, 48, 49, 50]. These natural paradigms underscore the advantages of integrating multiple dynamic responses into synthetic materials. Synthetically, incorporating multiple stimuli helps overcome individual limitations and enables more precise control over outcomes [51, 52]. These responses could be competitive, synergistic, or orthogonal and showed enhanced stress relaxation, mechanical strength, degradation, and self‐healing properties [53, 54]. Strategies for designing such systems are to carefully incorporate of extra stimuli to already present one [55], using double network polymer gels of different stimuli or using the same molecule for performing dual functions [56, 57]. One such example is incorporation of imine and boronate ester bonds in the polymer to get dual network hydrogel, that has shown not only dual dynamic behavior but also multiresponsivity to diols and reactive oxygen species apart from inherent pH and redox responsiveness [58]. Also, a coumarin derivative and a methacrylate derivative incorporated into a polymer showed effective photo and thermal responsiveness where sol to gel transformation was precisely controlled by UV and hydrophobicity by temperature. They also showed tunable cellular behavior on gel with respect to UV exposure duration and temperature [59]. By integrating DCvC principles, biomaterials can simultaneously address key requirements needed to create effective regenerative therapies, underscoring their potential to advance the field.

### 1.1 Regenerative Medicine: Scope and Material Requirements

Regenerative medicine encompasses a wide range of approaches aimed at restoring normal tissue function, from cell‐based therapies and bioengineered scaffolds to the delivery of growth factors and drugs that stimulate endogenous repair mechanisms [40, 60, 61]. Central to these approaches is the need for biomaterials that can support and guide the body's natural healing processes while seamlessly integrating into the host environment. However, designing such materials is a formidable task due to the complexity of the biological systems they must interact with [62, 63].

To be effective, materials for regenerative medicine should meet several stringent criteria (Figure 2):

1. **Biocompatibility**: [64, 65]. Materials must support cell viability and tissue formation, while avoiding triggering adverse immune or inflammatory responses, ensuring that they integrate harmoniously with surrounding tissues. This is particularly critical for scaffolds that interact directly with cells or are implanted long‐term.
2. **Adaptability**: [66, 67]. Regenerating tissues undergo dynamic changes in mechanical and biochemical conditions. Materials must adapt to these changes, for example, by adjusting their stiffness or degrading at a rate that matches tissue formation.
3. **Self‐Healing**: [68, 69]. Mechanical or structural damage to materials during their use in vivo can compromise their performance. Self‐healing materials, capable of autonomously repairing such damage, are especially advantageous in load‐bearing applications or environments subject to frequent mechanical stress; these materials are also amendable to minimally invasive procedures like injection and advanced biofabrication techniques.
4. **Cell Permissivity**: For biomedical application, cells need to repair during the course of healing and materials that allow cells to adhere, deform, and spread in its matrix or can pass through the cellular membrane to deliver the biochemical payloads, are of utter importance in regenerative medicine. For example, materials allowing cells to deform their matrix followed by relaxation during the cellular traction and promoting growth have proven beneficial. The kinetics of stress relaxation mediates cellular functions [70, 71]
5. **Degradability**: [72, 73]. Ideally, materials should degrade into nontoxic byproducts at a controlled rate, providing temporary support while allowing natural tissues to take over their function.
6. **Controlled Biofunctionality**: [74, 75]. Many regenerative therapies rely on the spatially and temporally controlled release of bioactive molecules, such as growth factors, cytokines, or drugs. Materials must be capable of delivering these agents with high precision to achieve optimal therapeutic outcomes.

**FIGURE 2:**
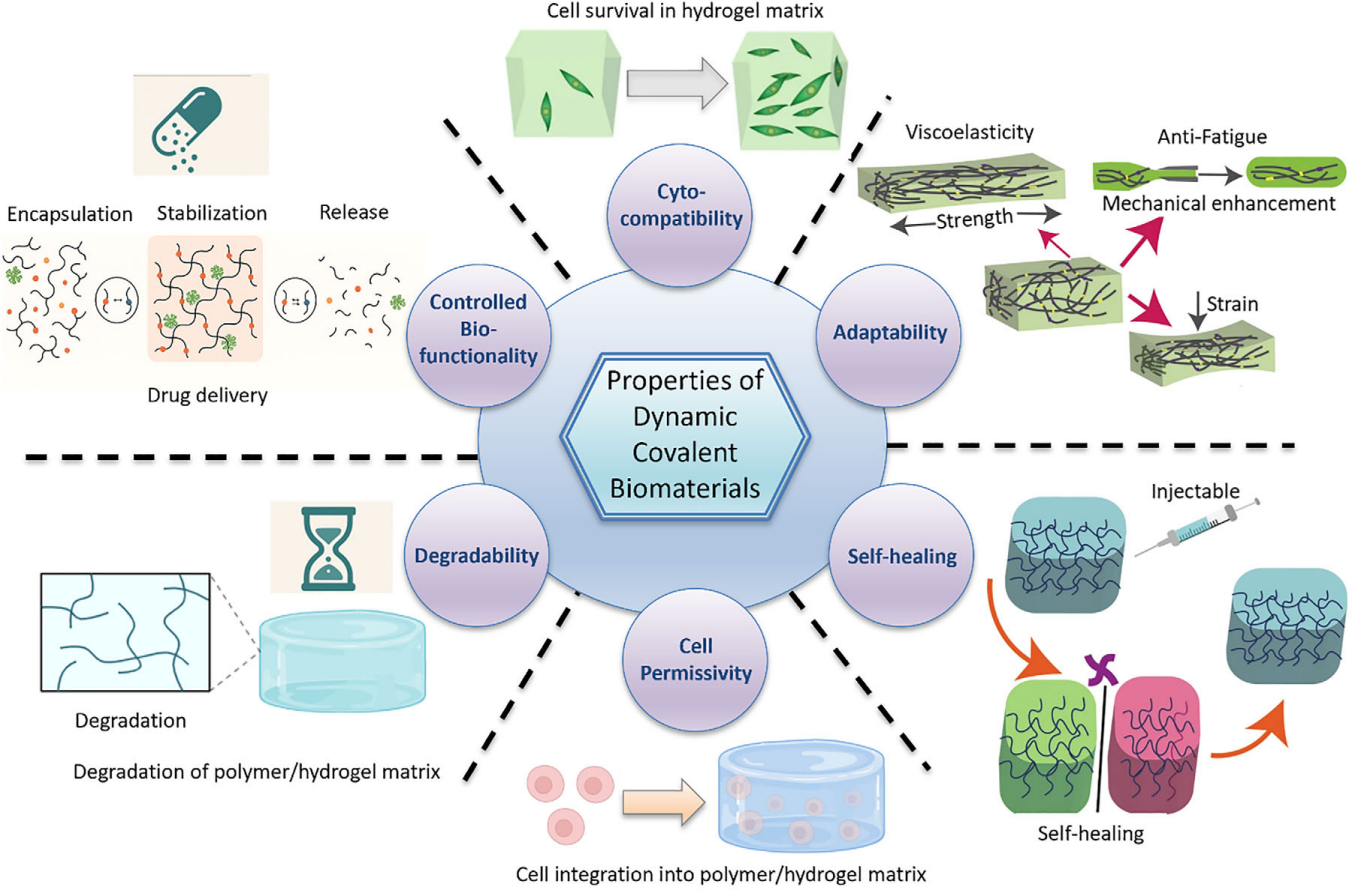
Key features of an “ideal” dynamic covalent biomaterial for applications in regenerative medicine. Illustrated are essential properties such as cyto‐compatibility, mechanical tunability degradation, release, and integration with biological functions to support tissue regeneration. Adapted with permission [44, 76]. Copyright 2023, Springer Nature.

Traditional biomaterials often fail to meet these complex and multifaceted requirements. The static nature of covalent biomaterials limits their adaptability and responsiveness, and while both synthetic and naturally derived biomaterials often have an inability to repair damage or dynamically modulate their properties that can lead to failure in demanding biological environments.

### 1.2 The Role of Dynamic‐Covalent Chemistry in Addressing Material Challenges

The reversible nature of DCvC‐based material allows them to continuously adapt to changes in their environment, mimicking the dynamic behavior of natural tissues [11, 12, 17], where covalent linkages are constantly broken and reformed to enable adaptation and maintain tissue homeostasis. In synthetic materials, dynamic covalent chemistry represents a first step toward mimicking this intrinsic adaptive capacity. For example, imine‐based hydrogels are pH‐sensitive, enabling their degradation or reassembly in response to the acidic conditions often found in inflamed or hypoxic tissues (chapter 6) [16, 17, 77]. Similarly, disulfide‐based materials can undergo reversible cleavage and reformation in redox‐active environments, making them ideal for applications in wound healing or drug delivery (chapter 3.3) [30, 78, 79].

Bonding kinetics has great impact on modulating the network temporal properties of the material that in turn governs the cell fate. Reversible bonding can enable a material to relax stress via bond breakage and reformation at crosslinking points. The field has clearly demonstrated that the relaxation of hydrogel matrix modulates stem cell proliferation and differentiation [80]. Chemically, reversible bond formation between polymer chains and crosslinker has been the tool to tailor stress relaxation in materials. The fast kinetics of bond formation and bond breaking by virtue of DCvC in imines, hydrazones, and boronate esters, is sufficient to enable the network to relax stress under cell‐generated forces, thereby allowing cells to remodel the matrix during tractions [81]. This stress relaxation arises by adaptability and remodeling of the network and molecular structure in the matrix due to dynamic covalent bonding.

The self‐healing properties of DCvC materials are particularly impactful in regenerative medicine [42, 82]. Scaffolds made from boronic ester‐based networks can repair microdamage caused by mechanical stress, maintaining their structural integrity and functionality over extended periods (chapter 3.2 and 6) [83, 84]. This capability is invaluable for applications in cartilage or cardiovascular tissue engineering, where materials are subjected to continuous mechanical loading.

Furthermore, the stimuli‐responsive nature of DCvC materials allows for precise control over therapeutic delivery [85, 86]. By designing materials that respond to specific biochemical cues, such as enzymes or metabolites, it is possible to achieve localized and on‐demand release of drugs or growth factors [87, 88]. This level of control enhances the efficacy of regenerative therapies while minimizing side effects.

This review aims to provide a comprehensive examination of DCvC's role in regenerative medicine, beginning with an in‐depth overview of the underlying chemical principles. This review supplants recent excellent reviews in the field which focus on the mechanoresponsiveness or mechanical design of dynamic covalent hydrogels [44, 81, 89, 90, 91]. The discussion will highlight the types of reversible covalent bonds commonly employed (chapter 3), their thermodynamic and kinetic properties (chapter 4), and their responsiveness to environmental cues, illustrating how these mechanisms enable the development of materials with exceptional adaptability, self‐repair, and biocompatibility (chapter 5). Building on this foundation, the review will explore current advances in DCvC‐based hydrogels, polymers, and hybrid biomaterials, with specific attention to innovations in tissue scaffolds, wound healing, and controlled drug delivery (chapter 6). This review will also examine how dynamic‐covalent chemistries intrinsic stimulus‐responsiveness allows precise control over material properties, enabling the development of systems that address critical challenges such as responding to mechanical stresses, dynamically adjusting degradation rates, and facilitating targeted therapeutic delivery. Furthermore, the review will extend this discussion by including more sophisticated and challenging multiresponsive systems. This transition is strongly inspired by nature, where multiresponsive behavior is fundamental to biological regulation. In this context, we will examine the bonding mechanisms, design strategies, and applications of such materials, with emphasis on how their spatiotemporal responsiveness governs material properties and directs cell fate. Finally, the review will conclude with a broader perspective on the field, outlining key challenges, future directions, and emerging opportunities.

## 2 Principles of Dynamic‐Covalent Chemistry (DCvC)

Incorporation of dynamic‐covalent chemistry into materials represents a paradigm shift in materials design by enabling the creation of systems that exhibit both the robustness of covalent bonding and the adaptability of dynamic processes [11, 12, 13, 92, 93]. Conventional covalent bonds, such as carbon–carbon (C─C) and amide (peptide) bonds are fundamental to the structural integrity of many organic molecules and biomolecules, like in proteins and DNA. However, their static nature limits their adaptability and responsiveness, posing challenges in designing adaptive materials. In Contrast, dynamic‐covalent bonds offer mechanism of reversibility and exist in a state of equilibrium (or trending to equilibrium) under thermodynamic and kinetic control [11, 94]. This reversibility is what sets DCvC apart, allowing molecules or networks to continuously reorganize, self‐heal, and adapt to changes in their environment.

Interestingly, dynamic‐covalent chemistry is not an entirely artificial construct but is ubiquitous in nature, where dynamic covalent bonds play critical roles in maintaining and regulating biological systems [95]. Disulfide bonds, formed between cysteine residues in proteins, exhibit reversible covalent bonding under redox control [28, 30, 96] are essential for the structural stability of proteins like antibodies while enabling conformational changes during processes such as protein folding or enzymatic activity. Another example of dynamic‐covalent processes in nature can be seen in DNA repair and recombination [97, 98, 99]. The cleavage and reformation of covalent bonds in the DNA backbone are mediated by enzymes like topoisomerases, which employ dynamic‐covalent interactions to resolve supercoils or repair strand breaks. This equilibrium ensures the genetic material remains intact yet accessible for replication and transcription.

Moreover, the dynamic formation and exchange of covalent bonds underpin the adaptability of natural materials, like extracellular matrix (ECM), which is critical for maintaining the structural integrity and functionality of connective tissues [60, 100]. In the ECM, reversible lysine crosslinking, in collagen, allow for tissue remodeling and repair in response to mechanical stresses or injury.

So, the presence of dynamic‐covalent principles in both synthetic systems and natural processes demonstrate their versatility and underscores the potential of DCvC to bridge the gap between artificial and biological systems, providing opportunities for biomimetic innovations in regenerative medicine, smart materials, and responsive drug delivery platforms.

## 3 Types of Dynamic‐Covalent Bonds

Dynamic covalent bonds represent a class of chemical interactions that combine the stability of covalent bonds with the reversibility typically associated with noncovalent interactions (Table 1) [11].

**TABLE 1:** Classification of common dynamic covalent bonds according to chemical structure, activation barrier/bond exchange energy, stimuli responsiveness, and reversibility.

Dynamic bond	Chemical structure	Typical activation barrier / bond exchange energy	Stimuli responsiveness	Reversibility	Refs.
Imine	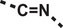	∼40–80 kJ mol^−^^1^	pH Heat (accelerate exchange)	**High**: Rapid reversible hydrolysis under mild acidic conditions.	[16, 17, 18, 23, 77, 101]
Hydrazone	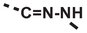	∼45–85 kJ mol^−^^1^	pH Heat (accelerate exchange)	**Moderate–High**: Reversible under acidic conditions but kinetically more stable than imines.	[19, 21, 102, 103, 104]
Oxime	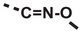	∼67–130 kJ mol^−^^1^	pH Heat (accelerate exchange)	**Moderate**: Slower bond exchange and greater hydrolytic stability than imines and hydrazones.	[22, 23, 105, 106]
Boronic ester	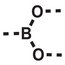	∼27–93 kJ mol^−^^1^	pH Diols Sugars ROS	**Very High**: Rapid and reversible diol exchange under aqueous conditions.	[24, 89, 107, 108, 109]
Disulfide	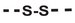	∼140–290 kJ mol^−^^1^	Redox agents UV Mechanical force	**Moderate**: Readily reversible via thiol–disulfide Exchange under redox conditions.	[28, 30, 79, 110, 111]
Diels–Alder	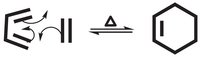	∼130–350 kJ mol^−^^1^	Heat Light (for photo induced reactions)	Moderate: Retro‐Diels–Alder occurs upon heating and reforms on cooling.	[37, 112, 113, 114, 115, 116]
Thia‐Michael	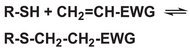	∼40–90 kJ mol^−^^1^	Heat pH catalyst light	**High**: Reversible Michael addition–elimination; dynamic exchange promoted by basic conditions and/or elevated temperature	[117]

### 3.1 Imines (Schiff Bases), Hydrazones, and Oximes

Imines, also known as Schiff bases, are formed through the condensation reaction between an amine and a carbonyl group, typically an aldehyde or ketone [18, 23, 101]. The reaction proceeds via a nucleophilic attack of the amine nitrogen on the electrophilic carbonyl carbon, followed by dehydration to yield the imine bond. The reaction is reversible in the presence of water, with the equilibrium position governed by the pH and the structural properties of the substrates (Figure 3).

**FIGURE 3:**
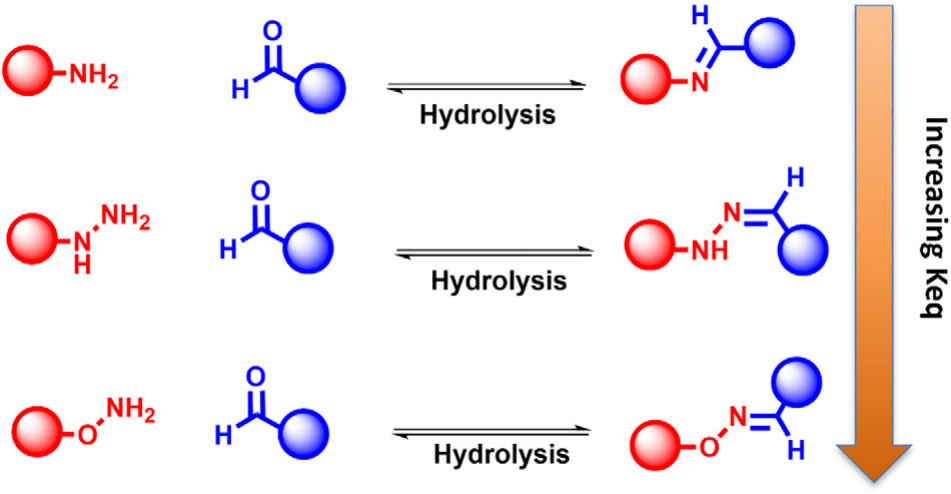
Dynamic covalent condensation of carbonyl compounds with amines, hydrazides, and hydroxylamines to form imines, hydrazones, and oximes. Adapted with permission [118]. Copyright 2023, Wiley‐VCH.

Hydrazones and oximes represent structurally similar dynamic bonds, formed when hydrazines or hydroxylamines react with carbonyl groups, respectively (Figure 3) [21, 22, 23, 104, 119]. Compared to imines, hydrazones and oximes exhibit enhanced stability due to the stronger electron‐donating properties via resonance of the nitrogen substituents, which reduce the electrophilicity of the resulting carbon–nitrogen double bond. The general mechanisms involve nucleophilic attack followed by dehydration, similar to imine formation. The reversibility of these reactions is highly pH‐dependent, typically favoring hydrolysis under acidic conditions.

Imines, hydrazones, and oximes are extensively used in hydrogel systems for tissue engineering and drug delivery [44, 120, 121]. For example, hydrazone‐linked hydrogels are employed for controlled drug release, where the degradation rate can be tuned via pH adjustments (chapter 6) [104, 122, 123]. Oxime chemistry has been exploited in cell‐adhesive scaffolds due to its high stability and biocompatibility, facilitating cellular integration and tissue regeneration [124, 125, 126].

### 3.2 Boronic Esters and Boronates

Boronic esters are formed by the reaction of boronic acids with diols, producing a boron–oxygen cyclic structure [24]. This reaction is highly dynamic due to the partial Lewis acidity of the boron atom, which readily undergoes exchange with competing diols or water molecules. Boronate complexes exhibit similar reactivity, often involving interactions between boronic acids and polyols or catechols [107]. The reversibility of these bonds arises from the equilibrium between the ester or complex and its unbound components, which is highly sensitive to pH and the electronic properties of the boronic acid.

The pH‐dependent formation equilibrium of a boronic ester in water was elegantly presented by the group of Pedro M. P. Gois in a 2019 review article (Figure 4) [24].

**FIGURE 4:**
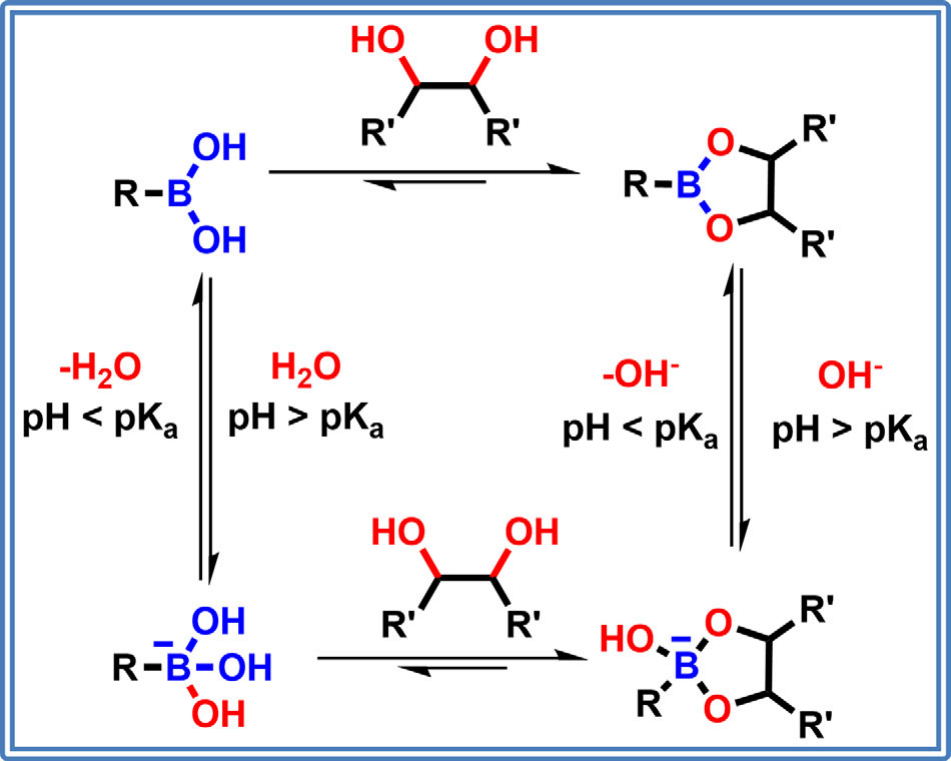
pH‐dependent formation of boronate esters from boronic acids and cis‐diols under aqueous conditions. Adapted with permission [24]. Copyright 2019, Royal Society of Chemistry).

The boronic ester bond formation mechanism involves the coordination of the diol hydroxyl groups to the empty p‐orbital of the boron atom, followed by dehydration. This process is reversible due to the susceptibility of boron to nucleophilic attack, allowing the dynamic exchange of diol partners.

Boronic ester‐based systems are widely utilized in glucose‐responsive materials for diabetes treatment [127]. Their ability to dynamically bind to diols in sugar molecules has been leveraged in glucose sensors and drug delivery systems [108]. In tissue engineering, boronic ester hydrogels have been designed to be pH‐responsive, enabling the controlled release of growth factors and the adaptation of mechanical properties to promote tissue repair (chapter 6) [108, 128, 129, 130].

In addition to the “classic” 1,2‐(cis)‐diol or 1,2‐dihydroxybenzene (catechol), other boronic acid ligands are also employed, some of which can respond to mild pH changes [118, 131]. A frequently used example is salicylhydroxamate, the hydroxamic acid derivative of salicylic acid (Chapter 6) [132, 133, 134, 135].

### 3.3 Disulfide Bonds

Disulfide bonds are covalent linkages formed between two thiol groups via oxidation [30]. The dynamic behavior of disulfide bonds arises from their susceptibility to reduction by reducing agents, such as glutathione, or oxidative cleavage in the presence of oxidants [136, 137]. The redox equilibrium enables the reversible formation and breaking of these bonds (Figure 5) [111].

**FIGURE 5:**
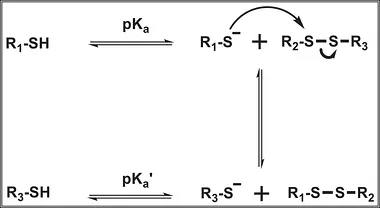
Schematic representation of the disulfide exchange mechanism. A thiolate attacks a disulfide bond, forming a new disulfide and releasing another thiolate. Adapted with permission [111]. Copyright 2014, Royal Society of Chemistry.

Mechanistically, the formation of disulfide bonds involves the oxidation of thiol groups, typically catalyzed by oxidative agents or enzymes [79, 138]. The cleavage of disulfide bonds, conversely, occurs via nucleophilic attack by thiolate anions under reducing conditions, leading to the regeneration of free thiol groups.

Disulfide bonds are prevalent in self‐healing hydrogels, where their redox‐responsive nature allows materials to adapt to oxidative stress or reducing environments (chapter 6). For instance, disulfide‐crosslinked hydrogels have been developed for the controlled release of therapeutics in environments with high glutathione concentrations, such as cancer tissues [78, 139]. Furthermore, their biocompatibility and responsiveness make them ideal for scaffolds in wound healing applications, where the redox conditions vary during the healing process.

### 3.4 Michael Additions

Michael additions are covalent linkages formed by the nucleophilic addition of a thiol or amine to an electron‐deficient α,β‐unsaturated carbonyl compound, such as acrylates, maleimides, or vinyl sulfones [79]. The dynamic behavior of Michael bonds stems from their ability to undergo reversible exchange reactions, typically via a retro‐Michael reaction under mild conditions such as elevated pH, temperature, or in the presence of excess nucleophiles (Figure 6) [34, 35].

**FIGURE 6:**
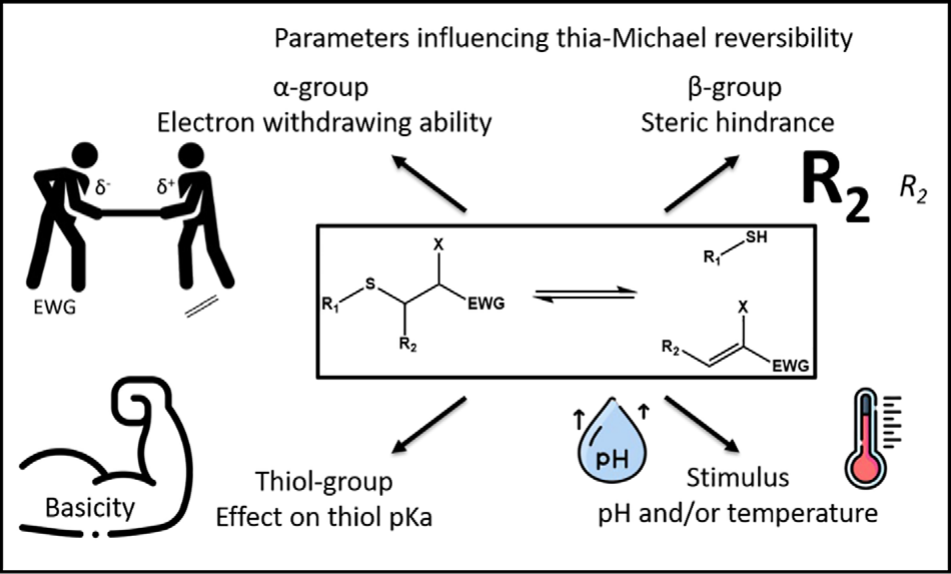
General reaction of a thiol with an electron‐deficient olefin via the thia‐Michael addition. The equilibrium of the thia‐Michael reaction is influenced by various factors, including pH, solvent polarity, temperature, and the electronic properties of the olefin and thiol. Reproduced with permission [140]. Copyright 2022, Multidisciplinary Digital Publishing Institute.

Mechanistically, the Michael addition involves a nucleophile attacking the β‐carbon of an activated double bond, resulting in the formation of a stable carbon–heteroatom bond. Reversibility is achieved when the leaving group (often a thiol) can dissociate, allowing for bond exchange with a competing nucleophile [35]. This equilibrium is particularly accessible in thiol–Michael systems and is influenced by the electronic nature of the Michael acceptor and environmental conditions.

Michael additions are increasingly used in dynamic polymer networks and hydrogels, where the reversible covalent bonding enables self‐healing, reshaping, and responsiveness to stimuli [141, 142]. For example, hydrogels based on reversible thiol–Michael linkages have shown promising applications in injectable systems or as wound dressings that conform to biological surfaces and can self‐repair (chapter 6).

In a biological context, Michael additions offer advantages due to their typically mild reaction conditions and potential for tunability. However, they also pose challenges. Nonspecific reactivity with endogenous thiols or amines in proteins may lead to off‐target modification, which can compromise selectivity and safety [79]. Moreover, the reversible nature of the bond may limit mechanical stability in long‐term or load‐bearing biomedical applications, particularly in environments where pH or redox conditions promote undesired bond exchange.

### 3.5 Diels‐Alder Reactions and Other Reversible Cycloadditions

The Diels–Alder (DA) reaction is a [4 + 2] cycloaddition between a conjugated diene and a dienophile, forming a cyclohexene adduct [113, 143]. The reversibility of the DA reaction—termed the retro‐Diels–Alder (rDA) reaction—enables dynamic behavior [144]. The equilibrium between the DA and rDA reactions is temperature‐dependent, with the rDA reaction favored at elevated temperatures due to the endothermic nature of bond cleavage.

Other reversible cycloadditions, such as 1,3‐dipolar cycloadditions, follow similar principles, involving the dynamic formation and dissociation of cyclic structures through changes in temperature, light, or chemical stimuli [145, 146].

Diels–Alder chemistry has been widely applied in the design of thermoresponsive hydrogels for drug delivery and cell encapsulation (chapter 6) [114, 147]. The reversible nature of the reaction allows for precise control of gelation and degradation, facilitating minimally invasive material deployment and controlled therapeutic release. Additionally, the DA reaction has been employed in tissue adhesives and coatings, where the reaction's specificity and tunable dynamics provide robust bonding while enabling material removal or reconfiguration under controlled conditions [118].

## 4 Dynamic Equilibria and Reversible Covalent Bonds

The behavior of dynamic‐covalent systems is fundamentally rooted in thermodynamic and kinetic principles, both of which play distinct yet interdependent roles in determining system behavior (Figure 7). Thermodynamically, the formation and breaking of dynamic‐covalent bonds are governed by the Gibbs free energy change (Δ*G*) of the system [148, 149, 150]. For a DCvC process to be reversible, the reactions must occur under thermodynamic control, meaning that the equilibrium state is determined by the minimization of Δ*G*. This property enables self‐correction: any deviation from equilibrium can be counteracted via reversible pathways, which is critical for features like self‐healing and adaptability [151].

**FIGURE 7:**
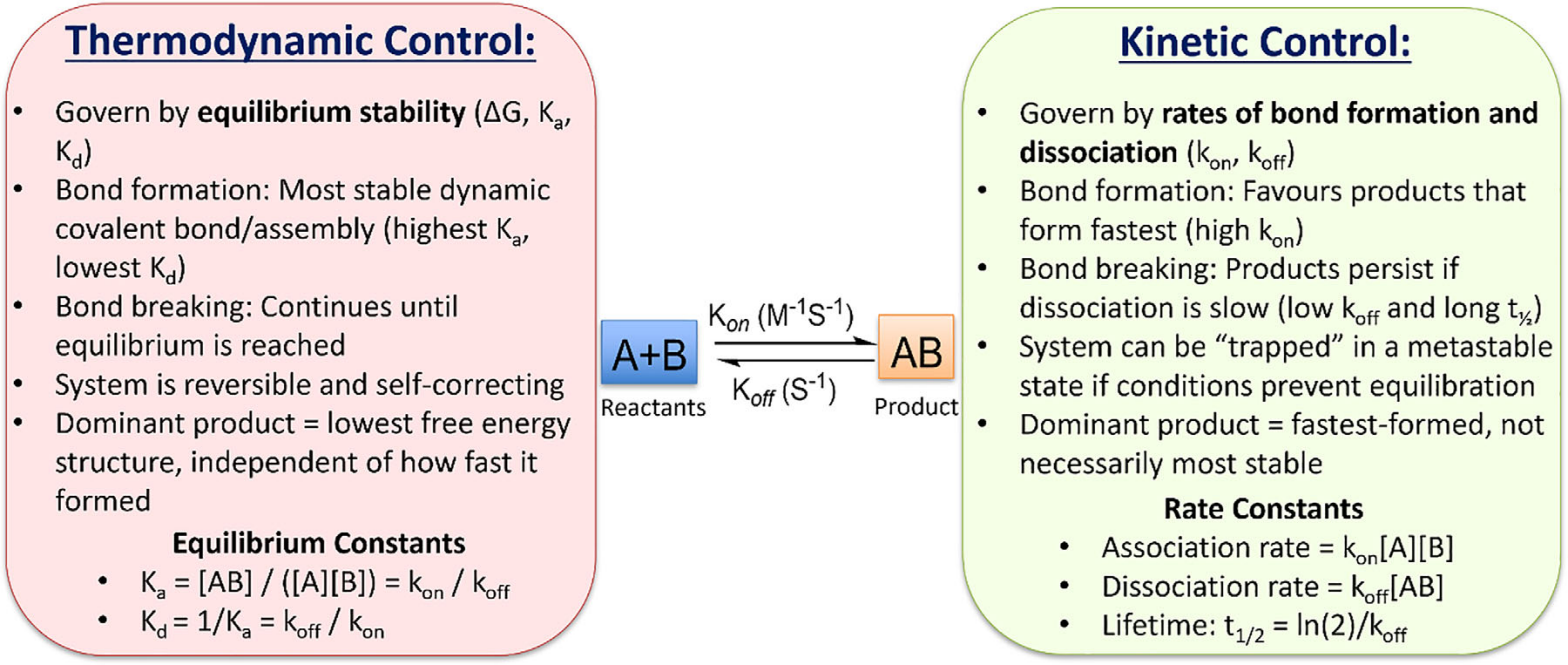
Conceptual understanding of Thermodynamic versus Kinetic control with respect to chemical equilibria.

However, modern understanding of DCvC systems show that they do not exclusively tends toward the formation of thermodynamic product only; numerous reports have shown that systems can be trapped in metastable states as well. Low energy barriers between most stable and less stable structures, transition state accessibility favoring less stable products under conventional conditions, fast assembly that trap the conformations in a locked state especially in case of multitopic precursors, and, extra stability and low solubility of kinetic or thermodynamic products are the common factors for encountering kinetic traps in the energetic landscape [152]. Liu et al. recently showed a polymeric system where the bias between thermodynamic versus kinetic self‐assembly of the polymer was based on the rate of water evaporation. Faster evaporation of water lead to kinetic trapped assembly (layered ionic network – semicrystalline), while slow evaporation led to thermodynamically favored amorphous cross linked network. Also, distinct material properties were observed for two materials (thermodynamic vs kinetic) [153]. Pausch et al. showed that subtle variations such as solvent choice or linker functionality can switch the system between kinetic and thermodynamic control. Topologically, low symmetry cages under kinetic control vs high symmetry structures under thermodynamic control were successfully shown. Low symmetry cages were precipitated out from the mixture giving no time for them to self‐correct into thermodynamic product [154]. Reports on imine‐based dynamic libraries show that, under certain conditions, they get locked into kinetically favored states, giving rise to supramolecular polymers that persist for long times. Even though the imine bonds are reversible, the system favors these metastable assemblies instead of equilibrating to the thermodynamic distribution [155]. In this regard, we can easily anticipate that this growing field needs more sophisticated experimental evidence and computational/mathematical tools to deduce the proper mechanistical understanding between thermodynamic and kinetic controls, especially for supramolecular and multitopic structures. These are significantly important to understand as the connection between molecular‐level parameters (rate and equilibrium constants, RECs), and bulk mechanical behavior is a common feature of dynamic material systems. Beyond governing gelation kinetics (∝ k_1_), network stiffness (∝ *K*_eq_), flow behavior (∝ k_−__1_ and *K*_eq_), and material stability or lifetime (∝ k_1_ and *K*_eq_), dynamic crosslinking interactions also play a critical role in regulating cellular interactions and in biomedical applications [101].

Although thermodynamics dictates the equilibrium state and composition, kinetics governs the rate at which equilibrium is reached [11, 16, 156]. This interplay between thermodynamics and kinetics is vital for tailoring the responsiveness of DCvC systems. Imines exhibit rapid exchange kinetics under mildly acidic or neutral conditions of bonds, making them well‐suited for applications requiring quick adaptability [157]. Like in bis‐aliphatic hydrazone formation kinetics varied from 3.9 ± 0.5 M^−^^1^s^−^^1^ at pH 5.3 to 15.5 ± 1.3 M^−^^1^s^−^^1^ at pH 7.3, while PEG–hydrazone gels underwent rapid degradation under acidic conditions but remained stable for ∼1.5 weeks near neutral pH [123, 158]. In contrast, bonds formed via reversible Diels–Alder reactions, which involve a cycloaddition between a diene and a dienophile, proceed with slower kinetics (*K*_app_ – 10^−4^ – 10^−3^ M^−^^1^s^−^^1^), providing greater stability while retaining reversibility [116, 159, 160]. These differences in kinetic profiles are critical for tailoring DCvC systems to specific applications [109]. Like drug delivery platforms often require controlled release over extended timescales, whereas tissue scaffolds may need to respond quickly to mechanical stress. Drug delivery systems should assemble rapidly through fast association kinetics, ensuring efficient complex formation even at equimolar reagent concentrations. Strong binding interactions are essential to stabilize the conjugate, while slow dissociation under dilution ensures sustained functionality. Tibbitt and coworkers showed network dynamics and binding equilibrium (*K*_eq_) directly affects the rate of cargo delivery. Cargo release increased with higher network dynamics (lower *τ*_r_ – faster relaxation) and reduced *K*_eq_ at elevated temperatures because accelerated bond exchange and weaker binding decreases the fraction of formed cross‐links, reducing network density and increasing mesh size, which in turn enhances drug diffusion through the gel [161]. Critically, dissociation must be accelerated in response to specific stimuli, enabling controlled cargo release or topology change for example from materials [162, 163]. Competitive diols such as fructose, mannitol, and glucose, together with temperature, altered the crosslink exchange dynamics in boronic acid hydrogels, resulting in time‐dependent changes in effective diffusivity and biomolecule release kinetics. Consistent with their competitive binding strengths, the equilibrium constants (*K*_eq_D) for fructose, mannitol, and glucose were found 40, 20, and 3, respectively, and the extent of drug release followed the same trend. These results demonstrate that dynamic topology evolution within the hydrogel network governs stimulus‐responsive transport and sustained release behavior [161].

The association and dissociation in DCvC systems follow second‐order reaction kinetics, described by the association rate constant (*k*_on_, units: M^−^^1^s^−^^1^), the dissociation constant (*K*_d_, units: M), and the dissociation rate constant (*k*_off_, units: s^−^^1^) [151, 164]. The half‐life of the complex (t_1/2_) can be calculated as: [165].

t1/2=ln(2)/koff

For practical applications, DCvC systems must operate within concentration ranges typical for biomolecular manipulations. For example, antibodies typically have stock concentrations of 0.1–1.0 mg/mL, corresponding to molarities of approximately 1–10 µM for standard antibodies (∼150 kDa). Smaller biomolecules, such as antibody fragments (∼50 kDa), proteins (20–100 kDa) or single‐domain antibodies (∼15 kDa), allow for working concentrations as high as 100 µM, while peptides can achieve even higher molarities. Based on these typical concentrations, achieving > 90% bound fraction is an important benchmark, which corresponds to a dissociation constant (*K*_d_) of approximately 1 µM under equimolar conditions. Although adhesive peptides are usually used in high concentrations, like in regenerative medicines the peptide concentration is often used as 1 mM [166]. For 1 mM peptide concentration, with >90% binding conditions, the dissociation constant (*K*_d_) corresponds to >100 µM.

For rapid assembly, a reaction time under 5 min is often desirable. Using second‐order kinetics, an association rate of *k*_o__n_ ≥ 300 M^−^^1^s^−^^1^ is required to achieve 90% binding within this time frame at a concentration of 100 µM.

The dissociation rate (*k*_o__ff_) is equally critical, as it determines the stability and longevity of the conjugate. The relationship between *k*_on_, *k*_o__ff_, and *K*_d_ is given by:

$$k_{off}=K_{d}\times k_{on}$$

For a system with *K*_d_ ≈ 1 µM and k_o__n_ ≈ 300 M^−^^1^s^−^^1^, the dissociation rate constant is approximately:

$$k_{\text{off}}=1\times 10^{-6}M\times 300M^{-1}s^{-1}=3.3\times 10^{-4}s^{-1}$$

The half‐life (*t*_1_/_2_) of the complex can then be calculated as:

*t*_1_/_2_ *=* ln(2) / 3.3 × 10^−^^4^ s^−^^1^ ≈ 35 minutes

Although this balance of parameters is suitable for some applications, it may be insufficient for delivery systems requiring extended stability, as premature cargo release can result in systemic toxicity. For pharmaceutically relevant systems, half‐lives on the scale of hours or days are typically required.

In Figure 8, various association rate constants for the formation of commonly used DCvC pairs are shown. It becomes evident that aldehydes and ketones exhibit very low association rate constants for condensation with hydroxylamines or hydrazides (10^−4^–10 M^−1^ s^−1^) [167, 168, 169, 170]. In contrast, thiols react much faster with maleimides via a thiol‐Michael addition (300 M^−1^ s^−1^) [169]. Even faster is the association rate constant for aryl boronic acids with catechols (1500 M^−1^ s^−1^) [170]. Via selection of chemistry, one can design materials platforms over 7 orders of magnitude with respect to the forward reaction rate.

**FIGURE 8:**
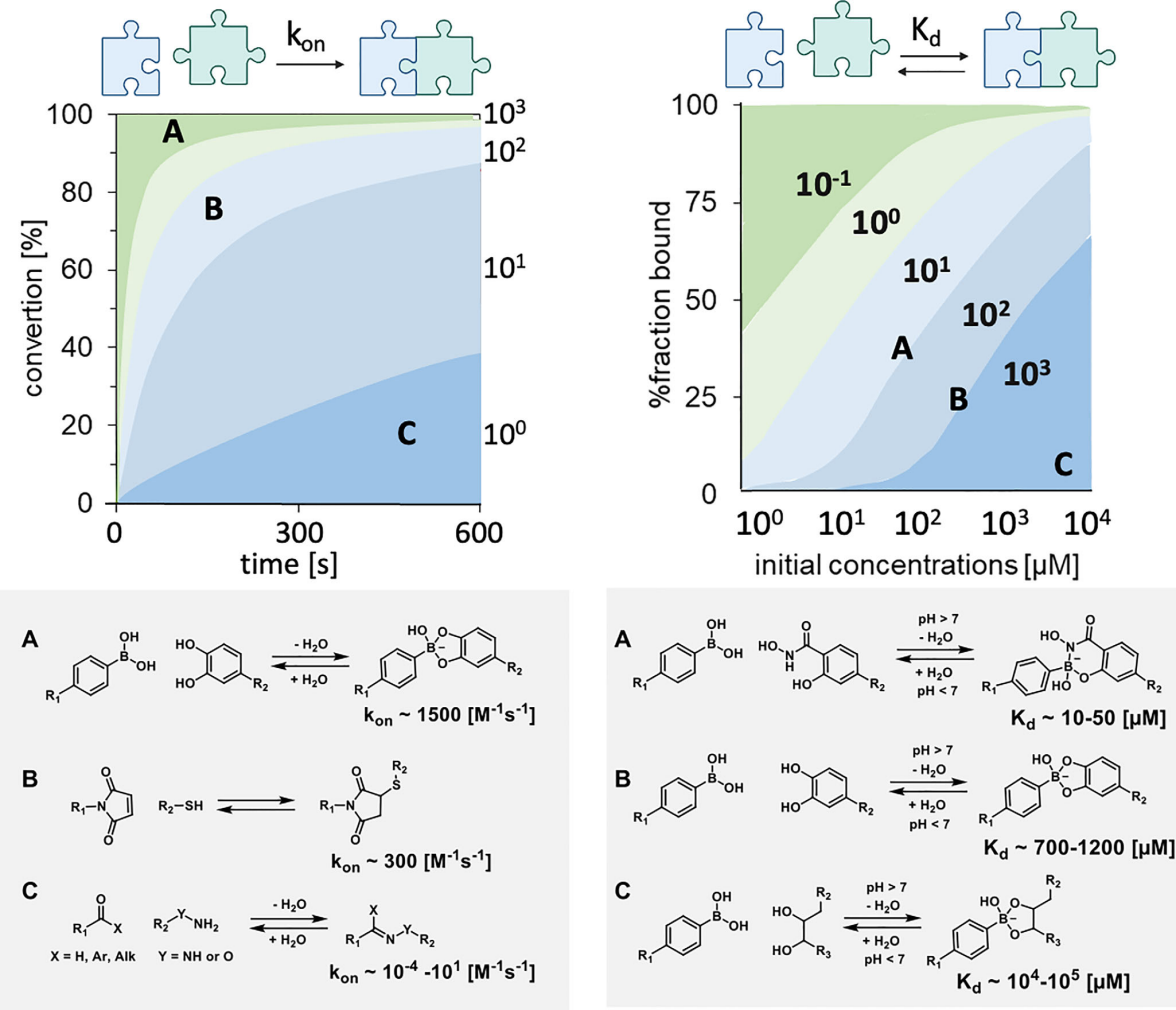
Left: Schematic illustration of the association rate constant (*k*_on_) for commonly used dynamic covalent chemistries, based on equimolar reaction progress as a function of time [167, 168, 169, 170]. Right: Apparent dissociation constants (*K*_d_) of different boronic acid derivatives — including cis‐diols, catechols, and salicylhydroxamates — shown as the fraction of bound species (condensation) plotted against the initial concentration of the binding partner [132, 171, 172].

A critical challenge in designing DCvC systems is balancing rapid assembly with prolonged, stimulus‐responsive dissociation. Enhancing dissociation rates by decreasing association rates (kinetics) often results in prolonged assembly times, while reducing *K*_d_ (thermodynamic) excessively can make the conjugate too stable, limiting reversibility. Important to emphasize, however, is that kinetics and thermodynamics capture different aspects of DCvC behavior. Association and dissociation rate constants describe how quickly complexes form or break, whereas the dissociation constant (*K*_d_) reflects their equilibrium stability. Thus, rapid binding does not necessarily imply strong binding, and conversely, a highly stable complex does not guarantee fast assembly. To illustrate this, Figure 8 schematically shows the dissociation constants of arylboronic acid complexes. It becomes clear that *cis*‐diols exhibit very high dissociation constants (10^4^–10^5^ µM) and are therefore only stable at high concentrations of the reactants [171]. Catechols, in contrast, reach at least the millimolar range (700–1200 µM) [172], while salicylhydroxamic acids still show dissociation constants in the low micromolar range (10–50 µM) [132].

Ultimately, innovative approaches that decouple these competing demands will be essential for advancing dynamic‐covalent systems in applications ranging from adaptive materials to drug delivery platforms.

## 5 Advantages of Dynamic‐Covalent Materials

Dynamic‐covalent materials (DCMs) are an innovative class of materials defined by their ability to form and reversibly dissociate covalent bonds in response to specific environmental conditions that align closely with the complex demands of regenerative medicine [11, 12]. The intrinsic dynamic behavior of these materials mirrors the dynamicity of biological systems, allowing them to integrate into regenerative contexts [14]. Below, we explore the fundamental advantages of DCMs in greater depth, emphasizing their multifaceted utility in biomedical applications.

### 5.1 Self‐Healing Capability

One of the most prominent features of DCMs is their self‐healing capability, which arises from the reversible exchange of covalent bonds that enables them to autonomously repair damage, such as cracks or structural disruptions, restoring their mechanical integrity without the need for external intervention [14, 173, 174]. Dynamic bonds with lower activation energies tend to heal faster and the activation barriers are generally lower than those of static covalent bonds [175]. Boronic esters show the fastest exchange due to low activation barriers (27–90 kJ mol^−1^), while imine/hydrazone/oxime systems are intermediate (40–130 kJ mol^−1^) and disulfide (140–300 kJ mol^−1^) and Diels–Alder bonds (130–350 kJ mol^−1^) require higher activation and heal more slowly. In contrast, static covalent bonds have much higher dissociation energies (300–600 kJ mol^−1^), making them effectively irreversible under self‐healing conditions.

For example, materials based on imine (Schiff base) chemistry exhibit self‐healing behavior in the presence of water or under slightly acidic conditions see chapter 3.1) [16, 17, 176]. This ability allows tissue‐engineered scaffolds to recover from mechanical deformation or microstructural damage, maintaining their functionality over extended periods.

Similarly, disulfide‐based materials leverage redox‐sensitive dynamic bonds to facilitate self‐healing in reducing environments (chapter 3.3) [30, 111]. This mechanism is especially beneficial in inflamed tissues, where the redox potential fluctuates, and the material must dynamically adapt while preserving its structural properties despite exposure to challenging biological conditions [110, 136, 177, 178].

Moreover, self‐healing properties are advantageous for minimally invasive medical procedures and processing. Injectable DCvC‐based hydrogels can be delivered as a liquid and subsequently form a cohesive, self‐healing scaffold at the target site [44]. This not only facilitates precise placement but also ensures the long‐term durability of the material under repeated mechanical stress, as is often required in cartilage or bone repair applications. In some advanced processing methods like 3D bioprinting, self‐healing also provides a decided benefit. For example, upon the application of shear, many DCvC materials rupture and shear‐thin, facilitating deposition via a 3D printing nozzle, while minimizing shear stress on cells [179]. Upon deposition, rapid or controlled self‐healing of the extruded material forms a solid scaffold via self‐healing of the material interface upon printing.

### 5.2 Adaptability to Environmental Changes

Another critical advantage of DCMs lies in their ability to dynamically adapt to environmental changes [92, 180]. Biological systems are inherently dynamic, with tissues and cells constantly exposed to fluctuating mechanical, biochemical, and physiological conditions [181, 182]. DCMs, can adjust their physical and chemical properties in response to such changes.

For instance, boronate ester‐based materials are highly sensitive to pH and ionic strength, enabling them to alter their stiffness or swelling behavior in response to local environmental conditions such as in wound healing application (chapter 3.2) [107, 129, 183]. Similarly, cartilage repair applications often benefit from hydrogels that can dynamically mimic the stiffness of native cartilage, ensuring compatibility with surrounding tissues and facilitating seamless integration (chapter 6) [103, 184, 185].

Dynamic covalent systems based on Diels–Alder chemistry provide another compelling example of environmental adaptability (chapter 3.5) [147, 159, 186]. These systems exhibit thermoresponsive behavior, allowing materials to adjust their crosslinking density and mechanical properties with temperature changes. Diels–Alder‐based scaffolds can remain liquid at ambient temperatures for easy delivery and transition into a stable, supportive gel structure at body temperature [187].

DCMs rely on reversible bonding to dissipate mechanical stress, a principle also relevant in biology where cells probe matrix and adapt to its viscoelasticity [81, 188]. Cells generate traction forces through adhesions and experience resistive feedback, whose timescale depends on matrix viscoelasticity and dictates cellular behavior [81]. Mechanistically, cells deform the matrix through actomyosin‐driven traction forces transmitted via focal adhesions, and even forces in the pico‐ to nano‐ newton range can rapidly influence signaling and gene expression [189]. Hydrogels with fast stress relaxation often promote active cell behaviors, as shown by enhanced MSC spreading, proliferation, and osteogenesis in alginate gels [80], increased endothelial spreading and vascularization in elastin‐like protein–PEG gels, and greater neurite extension and maturation in faster‐relaxing protein‐engineered hydrogels [190]. In contrast slower relaxing hydrogels, showed chondrocyte phenotype and cartilage‐like matrix deposition, indicating that sustained mechanical resistance can help maintain tissue‐specific function [191]. Crucially, these relaxation dynamics can be decoupled from bulk stiffness using dynamic covalent chemistries (e.g., hydrazone, imine, or boronate ester crosslinks), which allow independent control of the relaxation timescale without altering the initial modulus [192]. Additionally, the adaptability of DCMs plays a crucial role in controlling their degradation rates [72, 123]. By carefully designing the dynamic covalent chemistry, these materials can degrade at rates tailored to the specific needs of the regenerative process. For example, in tissue engineering, a scaffold may need to provide structural support during the initial stages of tissue formation but gradually degrade as new tissue matures and replaces the material [39, 193].

### 5.3 Stimuli‐Responsiveness

The stimuli‐responsiveness of DCMs represents one of their most versatile and powerful attributes [85, 121, 194]. This property enables precise spatial and temporal control over material behavior, allowing DCMs to interact dynamically with their environment or respond to specific triggers. Additionally, using the multistumuli strategy enhances the desired properties with more control over physiochemical parameters. Stimuli can be classified as endogenous (arising from within the biological system) or exogenous (applied externally), offering diverse opportunities for tailoring material properties to therapeutic needs [87, 195, 196].

#### 5.3.1 Response to Endogenous Stimuli

DCMs are designed to leverage physiological signals such as pH, redox gradients, or enzymatic activity to modulate their properties in response to local biological conditions [196, 197]. This can be one or multiple signals at a time depending on their chemical structure. pH‐sensitive DCMs, such as those containing imine, hydrazone, or boronate bonds, selectively degrade or release encapsulated drugs in acidic environments [17, 183]. This feature has been extensively explored in cancer therapies, where the acidic tumor microenvironment (approx. 6.5) triggers the release of chemotherapeutic agents, reducing systemic side effects and enhancing treatment specificity [135, 198, 199]. This approach was further advanced to multiresponsiveness, where to model the tumor microenvironment, a dynamic hydrogel based on dual dynamic covalent crosslinking, both boronate and imine cross links was designed. The hydrogel showed formation of stable tumor spheroids with mechanical effects of the gel impacting early growth, while the longevity of the system allowed spheroid stability up to 4 weeks [200]. Similarly, chronic wounds, which often exhibit an acidic pH ranging from 5.5 to 7.4, may benefit from such pH‐responsive systems [201, 202]. Such systems not only enable pH‐responsive drug release but, through the incorporation of dual dynamic networks, also exhibit mechanical adaptability by dissipating stress more effectively, thereby enhancing both structural resilience and bioactivity [200]. In this context, DCMs could enable the targeted release of antimicrobial or regenerative agents, thereby improving therapeutic efficacy and promoting tissue repair.

Redox‐sensitive materials, such as those incorporating disulfide bonds, respond to the elevated levels of reducing agents like glutathione found in inflamed or cancerous tissues (chapter 3.3 and 6) [43, 203, 204]. This property has been particularly useful in designing nanoparticles and hydrogels that deliver therapeutics directly to target sites, minimizing collateral damage to healthy tissues [205, 206, 207].

Enzymatic responsiveness further enhances the functionality of DCMs in regenerative medicine [13, 60, 208]. Materials containing enzymatically cleavable dynamic bonds can degrade selectively in response to enzymes overexpressed in pathological conditions. For example, hydrogels with matrix metalloproteinase (MMP)‐sensitive linkers degrade in the presence of MMPs, which are commonly upregulated in wounds or cancerous tissues [209, 210, 211]. This enzymatic degradation facilitates cell migration and tissue remodeling while simultaneously releasing bioactive agents or growth factors. Moreover, He et al. developed a double‐network hydrogel where a peptide network was reversibly cleaved and re‐ligated by enzyme transpeptidase, Sortase A (srtA) under mechanical load [212]. Basically, srtA first cleave the peptide bond synergistically in presence of mechanical force, between threonine and glycine to form acyl‐srtA intermediate, followed by peptide re‐ligation (by srtA) with glycine again, under removal of force. Since the enzyme decreases the activation barrier for cleavage, the force applied dictates the bond breaking at the enzymatic site. This force‐coupled enzymatic dynamic covalent chemistry enables injectability, self‐healing, and toughness without sacrificing stability. Such hydrogels showcase how enzyme‐mediated dynamic covalent bonds can deliver both robustness and adaptability, making them attractive scaffolds for regenerative medicine.

#### 5.3.2 Response to Exogenous Stimuli

Exogenous stimuli, such as temperature, light, electromagnetic fields, or mechanical force provide an additional layer of control over the behavior of DCMs [94]. Thermoresponsive hydrogels based on Diels–Alder chemistry transition between liquid and gel states with changes in temperature, making them ideal for minimally invasive applications such as injectable scaffolds (chapter 3.5) [213, 214].

Light‐responsive materials offer unparalleled precision, enabling spatially and temporally controlled changes in material properties [215, 216]. DCMs functionalized with photoresponsive moieties, such as azobenzene or spiropyran, can undergo reversible changes in crosslinking density, mechanical stiffness, or chemical composition when exposed to specific wavelengths of light [217, 218, 219]. This feature has been exploited in tissue engineering to create photocrosslinkable hydrogels, which solidify upon exposure to light, ensuring accurate scaffold placement and robust structural integrity (chapter 6).

Electrically and magnetically responsive DCMs, while less common, hold significant potential for specialized applications. For example, magnetoresponsive hydrogels have been used to guide cell alignment in neural regeneration, while electrically conductive DCMs have been explored for cardiac or neural tissue engineering, where electrical cues are critical for regulating cellular behavior [220, 221, 222].

Mechanochemical transduction has been long sought in biomaterials and control many applications like self‐healing, bond breaking, cell sensing the environment and delivery [56, 223]. Mechanical stimuli can be of various type such as compression, tension, shear stress, shaking, and hydrostatic pressures [224]. Dynamic covalent materials impart this response in the form of reversible bond breaking to dissipate tension because of the force and reform the bond to keep the integrity of the system. For example, mechanical force coupled with enzymatic activity of srtA enzyme to produce the dynamic hydrogel has shown successful mechanical reversibility [225]. A dynamic hydrogel of carboxymethyl cellulose having phenyl boronic acid and tannic acid was fabricated for the strain dependent stress relaxation. Apart from dynamic covalent boronic ester linkage they included additional noncovalent hydrogen bonding interaction which was also dynamic and showed strain‐enhanced rate of stress relaxation [226, 227]. They anticipated the cooperative effect of strain‐dependent breaking of both boronate ester crosslinks and interchain hydrogen bonds within the gel network as the underlying mechanism.

## 6 Design and Synthesis of Multistimuli Responsive Dynamic‐Covalent Materials

### 6.1 Dynamic‐Covalent and Supramolecular Interactions in Polymers

Dynamic‐covalent polymers are an emerging class of materials that combine the adaptability and reversibility of dynamic‐covalent chemistry (DCvC) with the robustness of polymeric structures [162]. The structural design of such polymers, including their architecture as linear or networked systems, and the possibility of incorporating physical interactions alongside dynamic‐covalent bonds, significantly influence their functionality and potential applic ations. This strategy leads to hybrid systems that combine the dynamic responsiveness of reversible covalent bonds with the versatility and tunability of noncovalent interactions [228]. Below, we explore these aspects in detail, focusing on how structural design and combined networks contribute to the unique properties of dynamic‐covalent polymers.

It is important to emphasize that not all crosslinked polymers are hydrogels. Networked polymers refer broadly to any polymer system featuring a covalently or noncovalently crosslinked structure.

Among the physical interactions used to complement DCvC, hydrogen bonding is one of the most extensively explored [92, 94]. Hydrogen bonds provide rapid and reversible self‐assembly and repair, complementing the slower but more stable nature of dynamic covalent exchange. A frequently utilized functional group in dynamic supramolecular systems is the 2‐ureido‐4[1H]‐pyrimidinone (UPy) moiety [228, 229, 230]. These units are known to form highly stable dimers through quadruple hydrogen bonding, with association constants typically ranging from 10^6^ to 10^7^ L·mol^−^^1^, making them particularly attractive for constructing robust yet reversible material networks [231]. Coupling supramolecular UPy interactions with imine‐based dynamic covalent networks (Figure 9A) creates dual dynamic polymer systems with synergistic control over network relaxation, mechanics, and adaptability. Moreover, this design offers a promising platform for future multiresponsive materials by combining the pH sensitivity of imine chemistry with the thermo‐ and mechano‐responsive nature of UPy assemblies.

**FIGURE 9:**
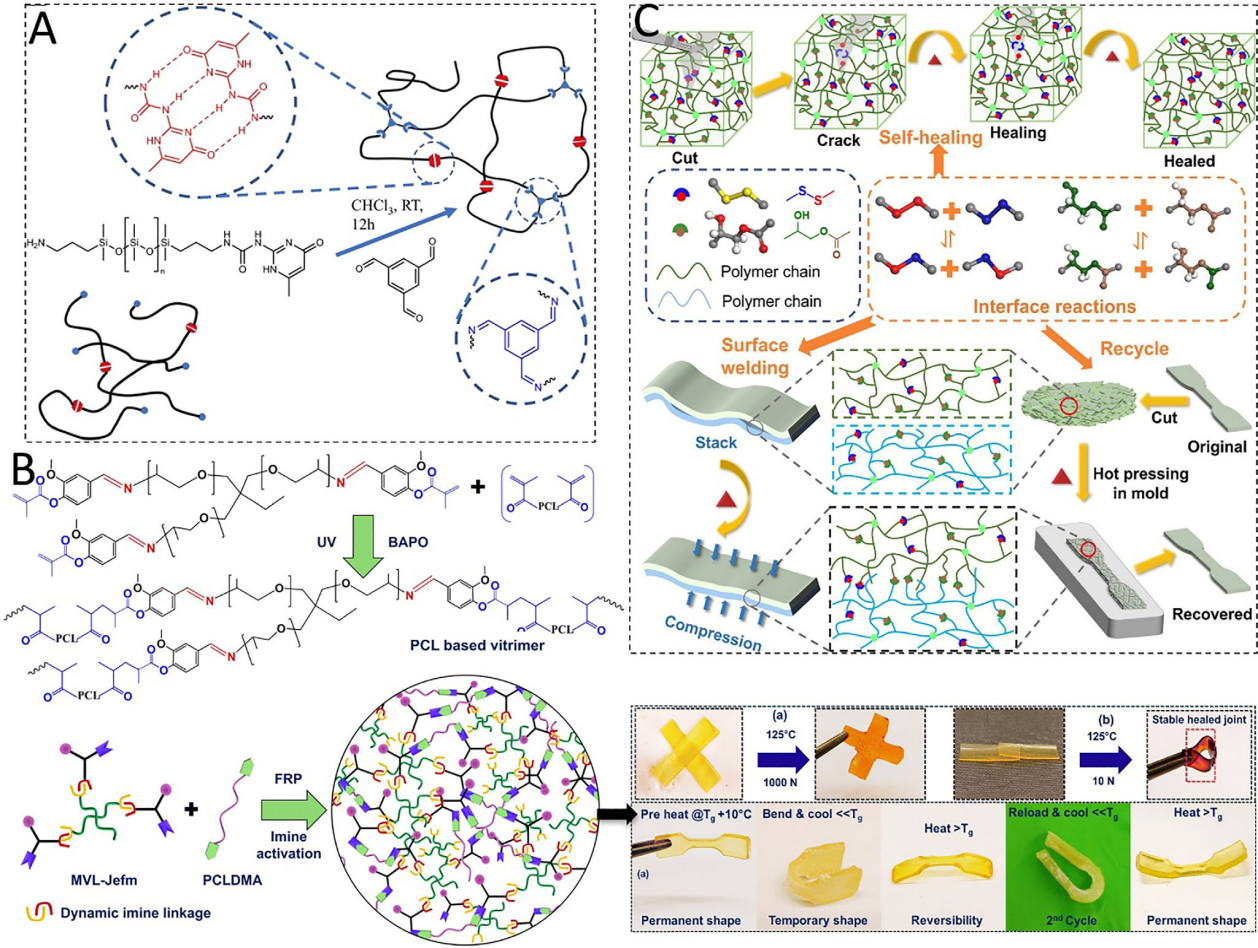
(A) Double‐network dynamic polymer with supramolecular (UPy‐based) cross‐links. Reproduced with permission [227]. Copyright 2023, Springer Nature. (B). Synthetic scheme and digital pics of PCL based vitrimer for self‐healing, welding and shape memory characteristics. Reproduced and adapted with permission [243]. Copyright 2025, Royal Society of Chemistry. (C) Dual dynamic bond mediated mechanism of surface welding, self‐healing, and reprocessing. Reproduced with permission [232]. Copyright 2024, American Chemical Society.

Hydrophobic interactions represent another powerful mechanism for reinforcing DCvC‐based polymers. In aqueous environments, the tendency of nonpolar moieties to aggregate strengthens the material's internal cohesion [119, 233, 234, 235]. Supramolecular interactions, including host–guest chemistry, further expand the capabilities of hybrid networks [236, 237, 238]. For instance, cyclodextrin‐based host–guest systems combined with other crosslinking methods yield materials with dual responsiveness to temperature (with phase transition windows typically between 25°C–45°C for aqueous systems) and chemical stimuli such as competitive guest molecules or changes in solvent polarity [239]. Cyclodextrin–guest interactions are strong and reversible, with binding constants typically around 10^2^–10^5^ M^−^^1^, enough to keep the structure intact with dynamicity. Increasing the amount of cyclodextrin in the polymer can raise the effective crosslinking by about 2–10 times, which makes the material more responsive, with temperature‐triggered changes shifting by roughly 20°C–30°C. These systems respond quickly to changes, usually within seconds to minutes, due to fast inclusion complex kinetics. By integrating both covalent and physical mechanisms, these hybrid materials achieve a level of complexity, adaptability, and biological relevance that surpasses what either system could offer alone, making them well‐suited for regenerative medicine and other advanced biomedical applications [240, 241].

The group led by Prof. Srivastava has developed a 4D‐printable, biodegradable poly(ε‐caprolactone)‐based vitrimer scaffold that leverages dynamic imine exchange to achieve shape‐memory, self‐healing, and cell‐adhesive properties without relying on supramolecular interactions [242, 243]. By incorporating methacrylated vanillin into PCL dimethacrylate networks, they created a flexible (glass transition temperature, 17.5 ± 2.2°C) and scalable material with excellent shape fixity (91 ± 1.7%) and recovery (92.5 ± 0.1%) at physiological temperatures (Figure 9B). The activation energy for imine exchange was found to be 48.0 kJ mol^−1^ in synthesized virtimers, which was lower than similar work in the past. Their design also promotes cell adhesion and reduces oxidative stress (0.22 of best virtimer in comparison with neat PCL scaffold having 1 ROS level), underscoring its promise for advanced applications in regenerative medicine and implantable devices. Yang et al. reported a vitrimer‐based soft actuator via associative dynamic covalent bonds that undergo reversible exchange, enabling reshaping, stress relaxation, and self‐healing without loss of crosslink density. Incorporation of carbonized silk fabric (CSF) provides photothermal and electrothermal conversion, allowing stimuli such as heat, light, electricity, solvent, and electromagnetic waves to trigger bond exchange. Damage healing was demonstrated by applying a voltage of 8 V, IR of intensity 0.56 W/cm^2^, and microwave of 800 W, where all the healing were based on temperature driven transesterification. The CSF‐vitrimer was highly conductive with a resistance of 29 Ω that became nonconductive on 100% stretching, but gained partial conductivity back on returning to original shape (80Ω) due to a shape memory effect. This synergy between bond chemistry and filler function ensures mechanical robustness while granting recyclability, multiresponsiveness, and controlled actuation [244]. Further, vitrimer systems can be tailored for two dynamic covalent chemistries to have multi responsiveness as β‐hydroxyl ester exchange and disulfide bond exchange. The ester bonds provide robust crosslinking and thermal reconfigurability, while disulfide bonds introduce faster bond exchange and redox sensitivity. Dual dynamic bonds accelerated stress relaxation to about 13.6 % faster than the single dynamic bond system, enable strong yet detachable adhesion with maximum adhesive strength reached to 18 MPa which was higher than the commercial adhesive Loctite (13.3 MPa) for steel surface. The material also showed repeated reprocessing with >90 % recovery of tensile strength after 3 cycles and self‐healing rate of crack width (> 86 % repair) after 30 min heating, which was higher than the single bond system (78 % repair)—making the material both mechanically durable and highly adaptable (Figure 9C) [232]. Prof. Katja Loos and her team have recently reported a method for creating vitrimer‐like elastomers based on lipoic acid. Their approach involves (i) the ring‐opening polymerization of lactones, (ii) subsequent modification of the resulting polylactones with lipoic acid, and (iii) crosslinking induced by UV light. Incorporation of disulfide bonds imparts self‐healing and redox capabilities to the material under UV irradiation, contributing to an extended service life [245]. Chemically correlated, they found that upon increasing the amount of polylactone chains in the elastomer, strain at break was increased from 25% to 180% and also stress at break from 0.34 to 1.4 MPa. Prodrug polymeric delivery systems based on disulfide‐based copolymer was reported by Du et al. which was able to self‐assemble into nano carriers to trap the hydrophobic drug paclitaxel and showed dual responsivity toward pH and the reducing agent TCEP. Lowering the pH altered the vesicle size, from 103 to 25 nm, by shifting the hydrophobic–hydrophilic balance, while reducing agents triggered aggregate disassembly followed by drug release. In vitro studies further demonstrated drug conjugated nanoparticle‐induced cytotoxicity, highlighting their promise as anticancer drug carriers [246].

### 6.2 Hydrogels Based on Dynamic Covalent Chemistry (DCvC)

A hydrogel's resemblance to natural ECM has made them indispensable in regenerative medicine, where they serve as scaffolds for tissue repair, vehicles for drug delivery, and platforms for cell culture (Figure 10) [247, 248, 249]. Prof. Tina Vermonden recently published a nice review article on stimuli‐responsive hydrogels as “dynamic smart biomaterials” [121]. In this section, the fundamental gelation mechanisms of DCvC‐based hydrogels are explored, alongside their tunable and stimuli‐responsive properties, which collectively underscore their transformative potential in regenerative medicine.

**FIGURE 10:**
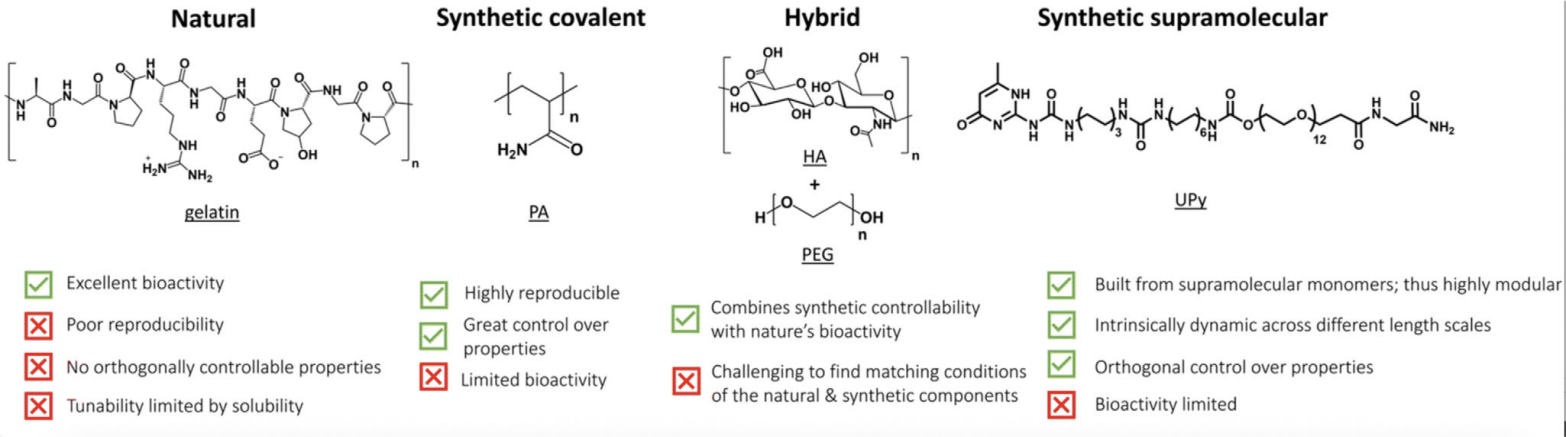
Advanced hydrogel systems developed to emulate the extracellular matrix (ECM), capturing essential chemical cues and mechanical properties of natural tissue microenvironments. Reproduced with permission [249]. Copyright 2024, Royal Society of Chemistry.

Chemical gelation is primarily mediated by specific types of reversible covalent bonds. Among these, imine bonds—formed through the condensation of aldehydes or ketones with primary amines— and imine‐type bonds, utilizing hydrazides or hydroxylamines, are among the most extensively studied (Figure 11A) [77, 119, 250]. Their pH sensitivity is particularly advantageous which enables their controlled degradation and sol–gel transitions, making imine‐based hydrogels highly attractive for applications such as drug delivery acidic microenvironments those found in inflamed, ischemic, or tumor tissues or tissue scaffolding around physiological pH

**FIGURE 11:**
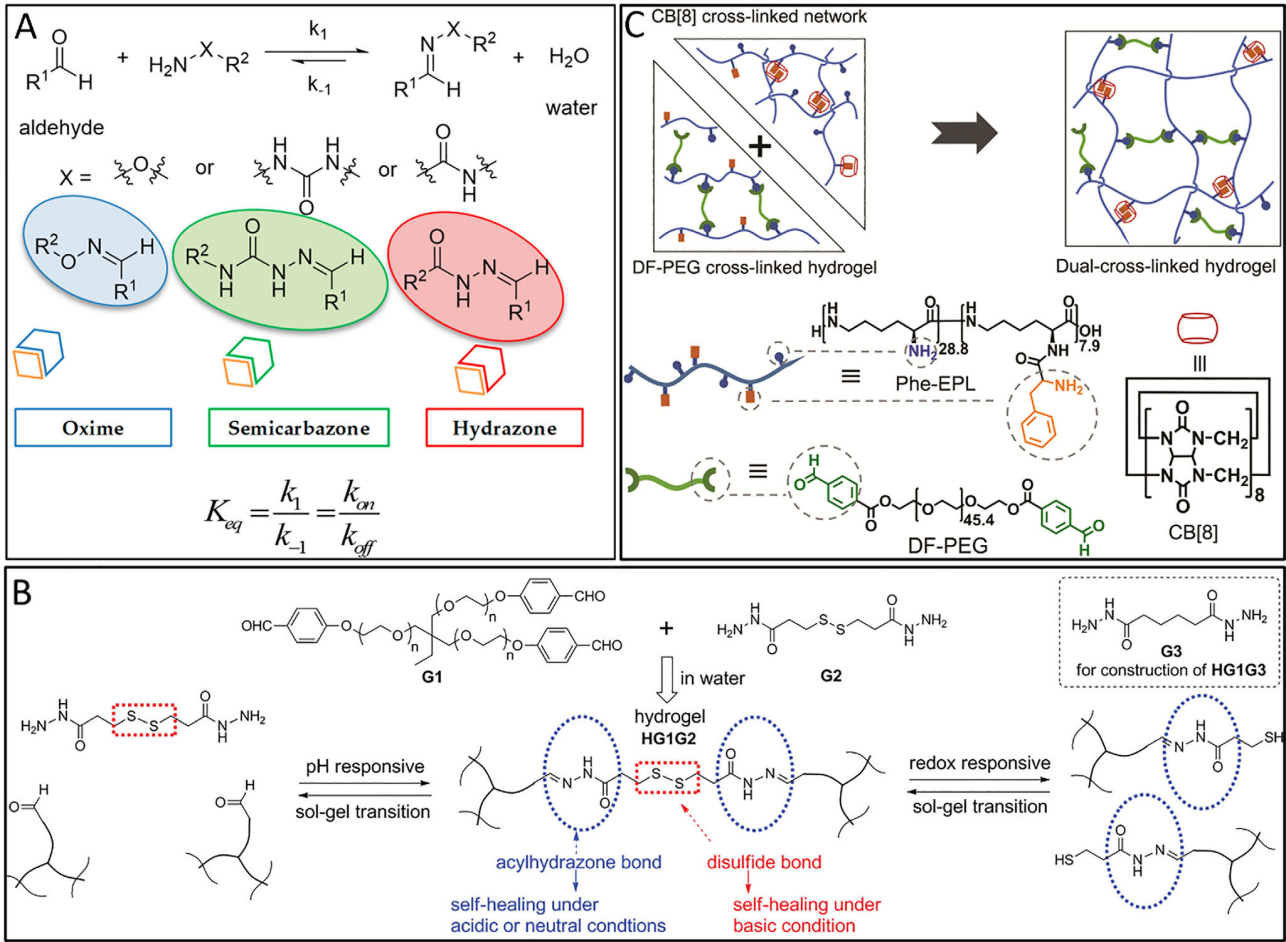
(A) Overview of reversible imine‐type bond formation. Reproduced with permission [119]. Copyright 2022, Multidisciplinary Digital Publishing Institute. (B) Design concept for a dynamic hydrogel exhibiting for self‐healing and dual sol–gel responsiveness, based on multiarm PEG bearing aryl aldehydes and disulfide hydrazides. Reproduced with permission [251]. Copyright 2012, American Chemical Society. (C) Chemical design of dual‐cross‐linked hydrogel with CB[8]. and imine linkages. Copyright 2019, Royal Society of Chemistry [252].

In efforts to tune the properties of single network hydrogels, researchers have integrated multiple orthogonal chemistries within one system, enabling more complex and adaptive responses than single‐bond networks. In 2012, Chen and coworkers reported dual‐responsive hydrogels containing both acylhydrazone and disulfide bonds (Figure 11B) [251]. They demonstrated that the orthogonal dynamic behavior of these two bond types enables the hydrogel to self‐heal effectively across a broad pH range—acidic, neutral, and basic—without the need for external stimuli at room temperature and in ambient air. Additionally, the hydrogel exhibited reversible sol–gel transitions triggered by both pH and redox changes, highlighting its potential for versatile applications requiring responsiveness to multiple environmental cues. In a recent example, we have shown how this orthogonal chemistry can be engineered together for invoking dual responsiveness for topological changes and drug delivery [253]. Doxorubicin was used as a cargo and as a crosslinking platform incorporating pH labile oxime and redox‐active disulfide bonds, which increases control and stability of cargo as compared to physical mixing. On applying either stimulus acidic pH or reducing conditions (TCEP), topological changes were observed until complete dissolution, but the drug remained bound to the polymer, as only one type of cross‐linking had been cleaved; however, the release of Dox was confirmed following the application of the successive stimulus. This dual pH/redox responsiveness was further used to fabricate cellulose based hydrogels, by Zhang and coworkers, with enhanced mechanical strength and as drug delivering carrier. They introduced a green catalyst called, 4‐amino‐DL‐phenylalanine (4a‐Phe) to enhance acylhydrazone formation and equilibriation. The hydrogel formation kinetics changed from minutes to seconds, on modulating polymer and catalyst concentration. The hydrogel was fully broken down under acidic conditions and re‐gelated upon neutralization with triethylamine, a process that was reversible over four cycles; additionally, it also showed redox‐responsive reversible sol–gel transitions, dissolving upon addition of the reductant 1,4‐dithio‐DL‐threitol (DTT) and re‐gelling when oxidized with H_2_O_2_. The hydrogel showed self‐healing (approx. 96%) and good biocompatibility by maintaining viability and proliferation of L929 cells [254]. Similarly, the excellent pH responsiveness of imine cross linking was coupled with dynamic host–guest interaction of cucurbit[8]uril (CB[8]) to form a double network hydrogel. CB[8]. based host–guest interactions are more dynamic than then imine linkage. The formed gel had tan *δ* value 0.60 (@ 1 Hz), for the dual network, which was higher than single network hydrogel (0.057 – imine crosslinks only), proving a large contribution to energy dissipation due to its fast dynamics. The system responded independently to two triggers: amantadine competitively disrupted CB[8] binding without collapsing the gel (modulus dropped drastically), while HCl cleavage of imine bonds converted it into a viscous solution. Applying both stimuli simultaneously led to complete gel disassembly into a free‐flowing liquid. The hydrogel also exhibited rapid self‐healing (Figure 11C) [252].

Another strong strategy for multi responsiveness for imine based crosslinking dynamic hydrogels is to integrate enzyme responsiveness for example using MMP sensitive collagen/gelatin biopolymers. This way the inherent pH responsiveness of imine crosslinking can be coupled with protein responsiveness and followed by tunable mechanical properties or degradation [255, 256]. In another study, Samanta and workers used a hydrazone‐crosslinked gallol functionalized hyaluronic acid (HA‐GA)‐based hydrogel to overcome the limitation of biomaterial implants against body's natural immune response. The hydrogel had dual, covalent (hydrazone) and noncovalent (H‐bonding by gallol) bonding and thereby showed enhanced mechanical properties. In addition to having inherent pH sensitivity, it also showed degradation in the presence of the enzyme hyaluronidase and showed radical scavenging activity, which makes it a dual responsive biomaterial. In vitro and in vivo studies indicated macrophage polarization toward an anti‐inflammatory phenotype, accompanied by suppression of proinflammatory cytokines [257]. In a recent example by Hao and coworkers, a multifunctional self‐healing hydrogel was designed for chronic diabetic wound treatment using an enzyme‐triggered cascade and dynamic imine bonds (pH sensitive). The hydrogel incorporated lysozyme, glucose oxidase (GOx), and catalase, providing antibacterial activity, glucose‐responsive pH modulation, and protection of the crosslinked network. GOx catalyzed the conversion of glucose into gluconic acid thereby lowering local pH to enhance imine crosslink density and self‐healing. The hydrogel also delivered rosuvastatin calcium to promote angiogenesis and resulting in accelerated healing of chronic diabetic wounds [258].

Another critical chemistry involves boronic ester bonds (Chapter 3.2), which are highly dynamic and exhibit remarkable sensitivity to pH and glucose concentrations [107, 183]. Under alkaline conditions, boronic esters can dissociate, enabling the hydrogel network to degrade or release encapsulated molecules. Additionally, their sensitivity to glucose has been exploited in glucose‐responsive systems for diabetic wound healing or controlled insulin delivery [130, 259, 260, 261, 262].

Boronic acid‐based hydrogels have been shown to remain stable in complex environments such as cell culture media and used for 3D cell culture applications. (Figure 12A) [84]. Notably, they also enable the formation of dynamic cocultures, supporting the simultaneous or sequential integration of different cell types.

**FIGURE 12:**
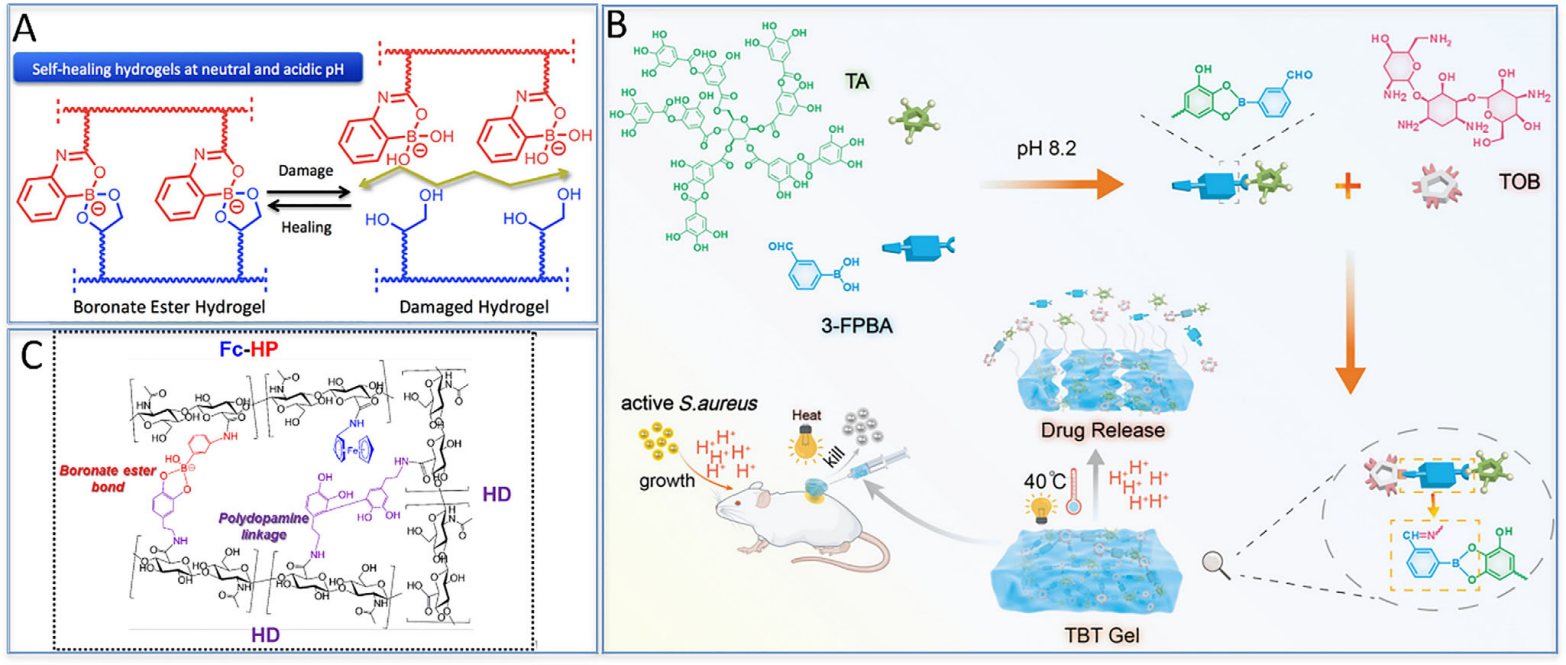
(A) Self‐healing properties of hydrogels formed from 2‐acrylamidophenylboronic acid and diol‐containing polymers under neutral and acidic pH conditions. Reproduced with permission [84]. Copyright 2015, American Chemical Society. (B) Schematic of dual network boronic acid hydrogel formation with pH and temperature sensitivity. Reproduced with permission [263]. Copyright 2022, Wiley‐VCH GmbH. (C) Design and crosslinking mechanism of hydrogel reactor. Reproduced with permission [264]. Copyright 2022, Elsevier B.V.

Boronate ester hydrogels have the ability to respond toward different cues making them ideal platforms to devise multiresponsive systems. Such as, boronic ester modified Alginate/PVA hydrogel showed sensitivity toward shear force and pH followed by application in drug delivery. A more stable interconnected network structure was formed at elevated pH, while, at low pH drug was released more quickly because of less stable sp^2^ boronate ester bonding. Also, the drug release decreases with increased shear rate amid less time for relaxation exhibiting force sensitivity of the hydrogel [265].

Extending the concept on multicomponent network designs to introduce additional functional outputs, a dual network based on bifunctional formylboronic acid was developed to simultaneously integrate antibacterial activity and multistimuli responsiveness, where both pH and thermal cues regulated drug release, while intrinsic catechol chemistry enabled H_2_O_2_‐triggered degradation (Figure 12B) [263]. Both in vitro and in vivo studies demonstrated that mild heating triggers drug release from the hydrogel, leading to strong antibacterial effects (Figure 12B). In an interesting example, hydrogel reactor was developed for local cancer therapy on ferrocene and GOx based dual network of phenylboronic acid–dopamine esters and polydopamine. The hydrogel was multiresponsive toward glucose, pH, and near‐infrared (NIR) light. GOx catalyzed glucose consumption to induce tumor starvation and generate H_2_O_2_, while ferrocene triggered Fenton reactions for chemodynamic therapy. The properties were validated in cancer where it led to significant tumor growth inhibition in vivo, demonstrating a versatile platform for multimodal cancer treatment (Figure 12C) [264].

Hydrogels based on disulfide bonds offer another chemical dimension for fabricating network architecture with selective responsiveness [136]. Disulfides participate in thiol–thiol exchange reactions (metathesis), allowing the hydrogel network to reorganize under redox conditions [266]. This redox sensitivity is particularly advantageous for applications such as cancer therapy, where tumors often exhibit an altered redox state, or for controlling the release of anti‐inflammatory agents in oxidative stress environments like in wound healing.

The group led by Prof. Grinstaff has developed on‐demand dissolvable hydrogels, offering a highly versatile approach to controlled material disassembly [178]. The four distinct chemistries employed for hydrogel dissolution—namely, thiol–thioester exchange, thiol–disulfide exchange, retro‐Michael‐type reaction, and retro‐Diels–Alder reaction—constitute a powerful synthetic toolbox. In an interesting example, Wang et al. synthesized hydrogel based on visible light driven thiol‐disulfide exchange. The hydrogel showed different mechanical properties with respect to light and also showed redox sensitivity in presence of DTT allowing degradation. In addition, the gels show rapid self‐healing, influenced by humidity and temperature, highlighting their potential as smart, multiresponsive biomaterials (Figure 13A) [267]. Noteworthily, di‐sulfide based covalent networks have inherent sensitivity for redox environment but also have been shown to possess light [268], temperature [269], and mechanical force [270], responsiveness, and hence are suitable for making multiresponsive materials. The idea of harnessing disulfide chemistry for mechanochemical responsiveness in hydrogels was shown by Lee et al., where PEG hydrogels crosslinked with disulfide bonds broke under compression or tension (> 30% for 30 s) to generate thiols which further reacted with a Michael acceptor (fluorescein‐5‐maleimide). They used this approach for force‐pattern immobilization of cell‐adhesion peptides, producing spatially defined areas where cells attach (Figure 13B) [271]. Building on this foundation, Huang and coworkers, developed double‐network disulfide hydrogels that respond to stretching, allowing the immobilization of fluorophores and peptides directly under mechanical load (Figure 13C) [272]. Together, these works highlight the inherent redox sensitivity of disulfide bonds, combined with mechanochemical activation, showcase a promising multiresponsive character that can be exploited for dynamic and adaptive biofunctions.

**FIGURE 13:**
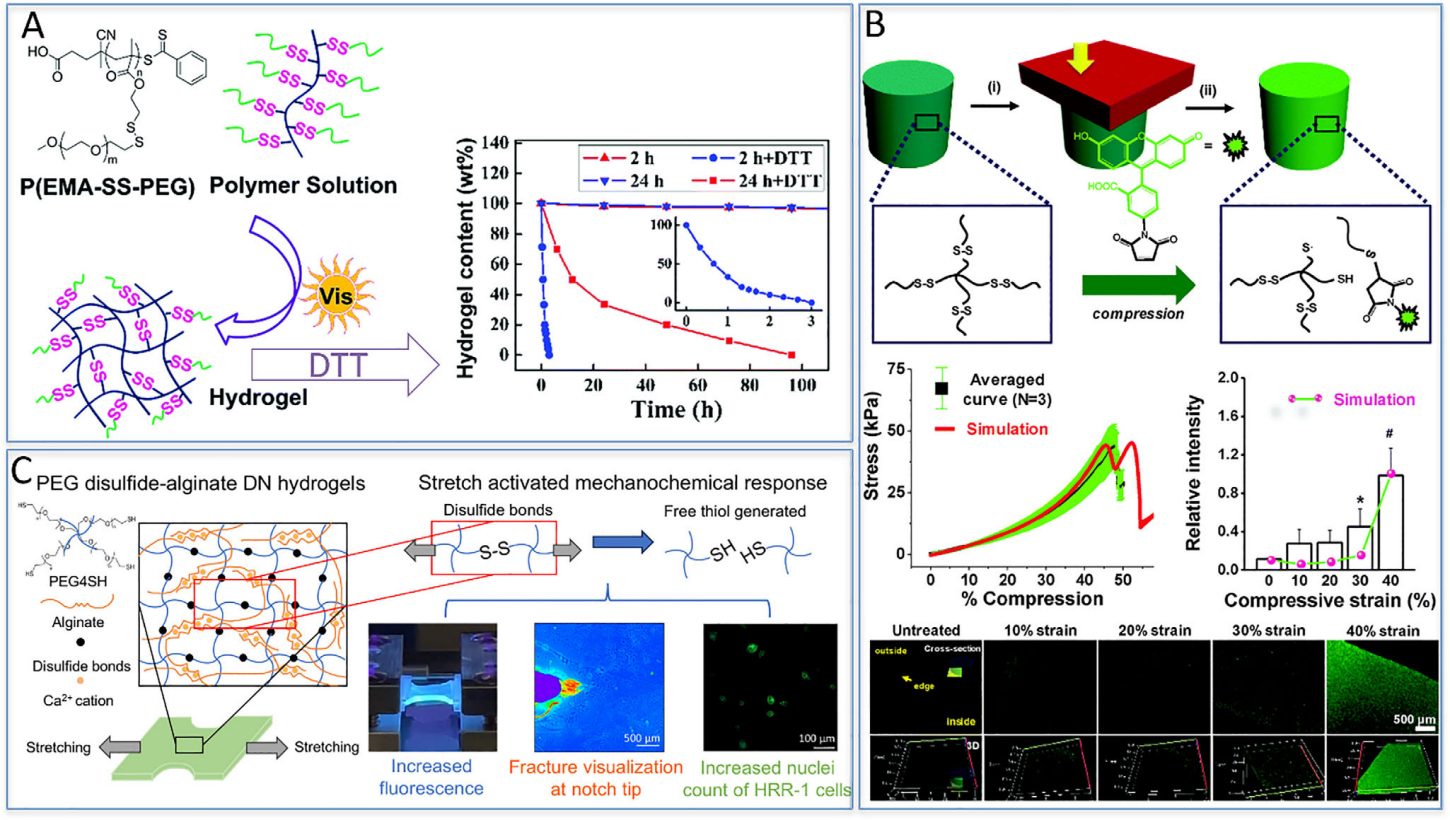
(A) Illustration for the hydrogel formation via visible light mediated thiol‐disulphide exchange and responsiveness to DTT. Reproduced and adapted with permission [267]. Copyright 2022, Royal Society of Chemistry. (B) Disulfide bond rupture by compression and subsequent Michael addition followed by Average stress–strain relationship and simulation for the gel in compression and fluorescence intensity of gels as a function of compressive strain under laser scanning confocal microscope. Reproduced with permission [271]. Copyright 2016, Royal Society of Chemistry. (C). Schematics of the hydrogel chemistry and disulfide bond breakage by stretch followed by reaction with fluorophore to give different responses. Reproduced with permission [272]. Copyright 2025, Elsevier Inc.

The Diels–Alder reaction offers a bioorthogonal and catalyst‐free approach to (dynamic) covalent hydrogel formation under mild conditions, making it highly suitable for biomedical and materials science applications [112, 273]. Another strategy to achieve Diels–Alder hydrogels involves modifying biopolymers with diene groups—commonly furan—followed by controlled crosslinking using bis‐maleimide linkers. Figure 14A illustrates various natural biomaterials functionalized with peptides or proteins that have been employed in this context.

**FIGURE 14:**
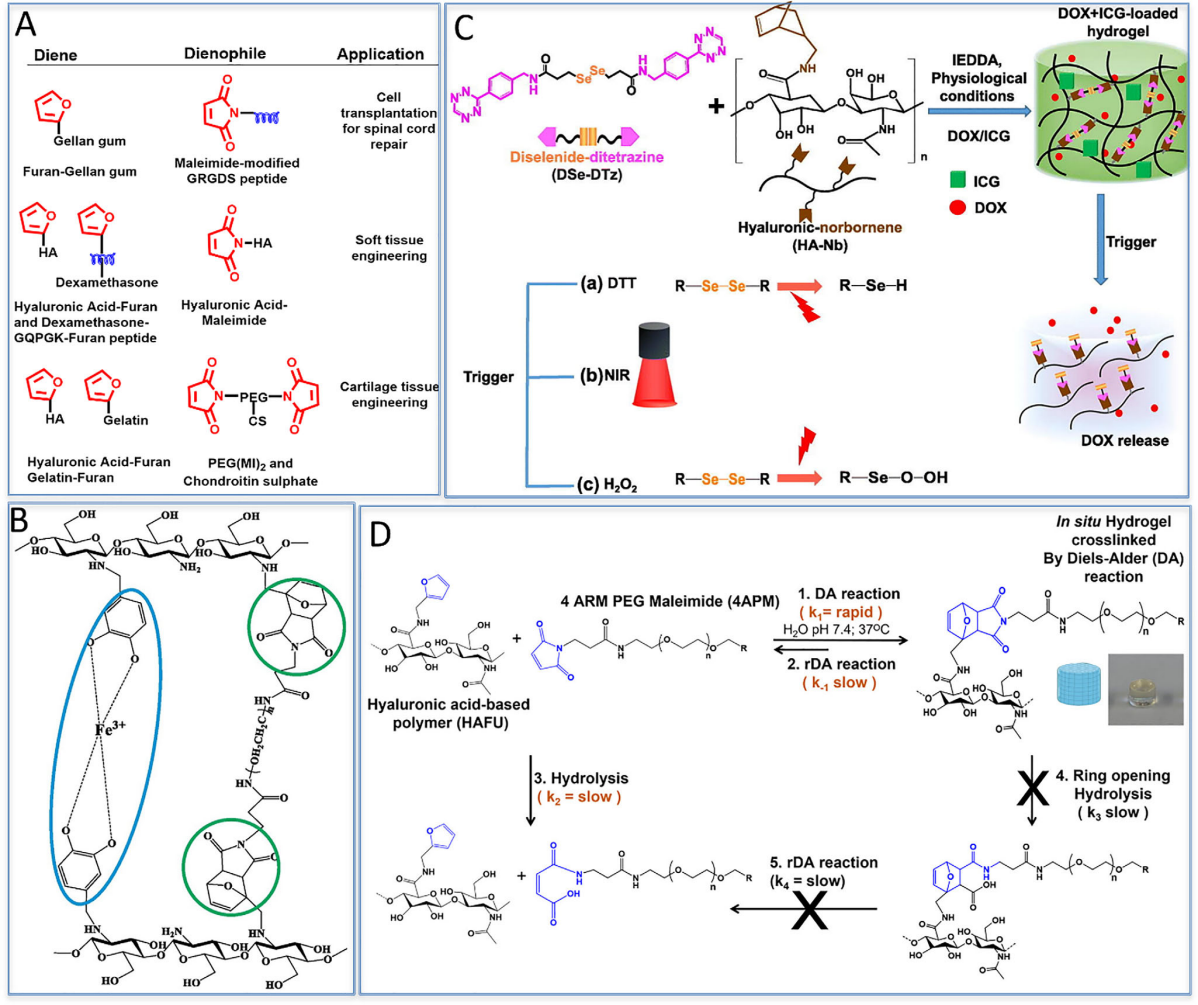
(A) Examples of biomedical hydrogels formed via Diels–Alder chemistry using various furan‐ and maleimide‐functionalized (bio)polymers as crosslinking partners. Adapted with permission [112]. Copyright 2020, Wiley‐VCH GmbH. (B) Schematic representation of dual crosslinked hydrogel networks combining reversible Diels–Alder linkages with catechol–Fe^3^^+^ coordination, constructed using N‐(furfural)‐modified chitosan. Reproduced with permission [274]. Copyright 2017, Elsevier B.V. (C) Synthesis of multi‐responsive hyaluronic acid–based hydrogels and the mechanism of diselenide bond cleavage under different stimuli for drug release. Reproduced with permission [275]. Copyright 2022, Elsevier Ltd. (D) Illustration of the DA‐based hydrogel crosslinking mechanism and possible degradation pathways. The system consists of six interconnected reactions forming a dynamic equilibrium, in which the first reaction occurs at a higher rate than the others. Reproduced with permission [187]. Copyright 2022, American Chemical Society.

Double cross‐linked hydrogels based on the Diels–Alder reaction and catechol–Fe^3^^+^ coordination have been developed using N‐(furfural) chitosan to form dynamic hydrogel networks (Figure 14B) [274]. Wang and coworkers demonstrated that adjusting the pH value can modulate the color, crosslinking mechanism, and mechanical properties of these dual‐network hydrogels. The multi chemical functional strategy was further explored via introduction of aldehyde and methacrylate groups into the HA backbone. This approach enabled dynamic Schiff base linkages to impart injectability, self‐healing, and tissue adhesion, while subsequent photoinitiated methacrylate polymerization locks in permanent covalent crosslinks for mechanical robustness. The cross linker also had the disulfide bond making it sensitive toward different stimuli. The hydrogel showed responses to mechanical force, UV‐light, and enzymes. It also showed good biocompatibility and tissue adhesive properties making it an attractive candidate for wound healing applications [276]. Diselenide based bonding in DA crosslinks would impart an extra stimuli response because diselenide has sensitivity towards oxidizing and reducing environment [277]. When diselenide was incorporated with inverse‐electron‐demand Diels–Alder (iEDDA) chemistry the resulting hydrogels showed multistimuli responsiveness to acidic pH, redox conditions (ROS/GSH), and near‐infrared (NIR) light through incorporated indocyanine green. The hydrogels showed fast and high swelling % >900 and showed controlled release of doxorubicin under tumor‐like conditions. These hydrogels demonstrated injectability, cytocompatibility, and enhanced anticancer efficacy, making the platform promising for cancer therapy (Figure 14C) [275]. Recent advances highlight how combining DA crosslinks with degradable backbones can yield hydrogels that integrate reversible covalent dynamics with biologically relevant degradation pathways. For example, Ilochonwu et al. employed hyaluronic acid–PEG networks formed by DA reactions for intraocular delivery of bevacizumab. In this system, the DA chemistry afforded tunable gelation and reversible stability, while hydrolytic degradation of the backbone governed long‐term release. Together, these studies illustrate how pairing DA dynamic covalent bonds with degradable polymers creates dual‐responsive hydrogels that are both mechanically adaptive and biologically aligned, opening new avenues for sustained, localized therapy (Figure 14D) [187].

Similarly, incorporation of photoresponsive groups, such as photoreversible covalent bonds or photosensitive linkages, hydrogels can be triggered to undergo structural or chemical changes with high spatial and temporal precision (Figure 15 A,B,C). For example, ultraviolet or visible light can induce the cleavage or rearrangement of specific covalent bonds, enabling the material to locally stiffen, soften, or degrade. This capability is particularly valuable in tissue engineering, where light can be used to sculpt hydrogel scaffolds in situ or release bioactive molecules at defined locations. A notable example involves disulfide bonds, which can undergo homolytic cleavage under UV irradiation to form thiyl radicals [79, 278]. These radicals can subsequently recombine, allowing the disulfide bonds to break and reconstruct in a random yet reversible manner—providing dynamic tunability to the material's network structure [278, 279, 280]. Li et al. developed a PEG‐based hydrogel incorporating both Diels–Alder adducts and disulfide crosslinks, enabling orthogonal mechano‐ and photo‐responsive chemistry. Mechanical stress triggered retro DA cleavage to reveal maleimides for thiol–Michael conjugation, while UV irradiation induced disulfide cleavage for additional functionalization. This dual‐stimulus design allows sequential and spatially resolved attachment of bioactive molecules, demonstrated by patterning different peptides to control multicellular adhesion (Figure 15 D,E) [281]. More recently, Liu et al. introduces a photocontrolled metallopolymer adhesive that enables reversible assembly of hydrogels through Ru–thioether coordination. Light irradiation cleaves the Ru─S bonds, allowing disassembly, while heating reforms the coordination to restore adhesion, thus providing an orthogonal glue–deglue cycle. By combining this adhesive with hydrogel building blocks engineered to be pH or thermoresponsive, the system demonstrates genuine multiresponsiveness (light, temperature, pH) within the same platform (Figure 15F) [282].

**FIGURE 15:**
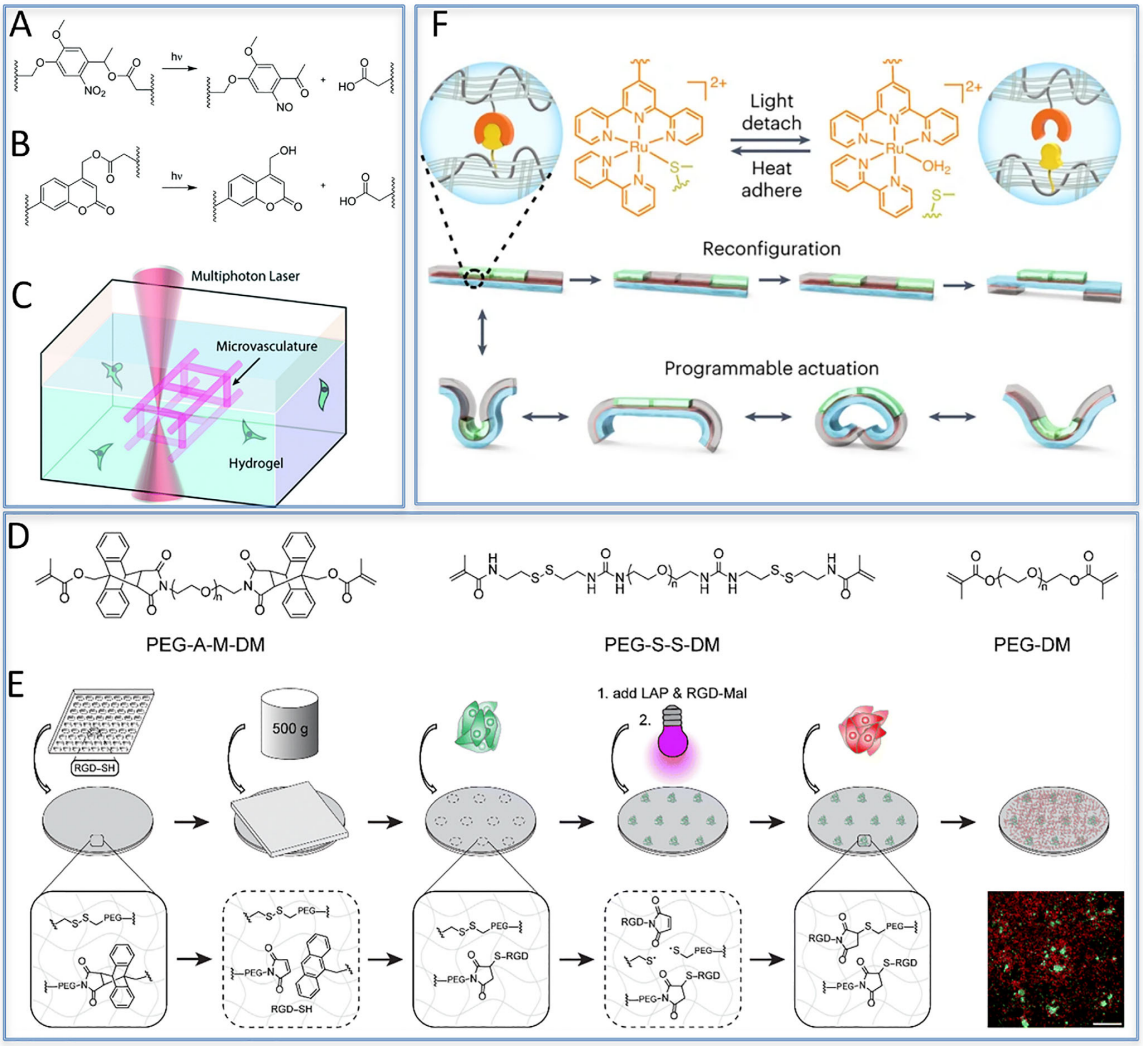
(A and B) Light‐triggered cleavage of photolabile groups such as nitrobenzyl and coumarin, leading to bond breakage when integrated into polymer networks. (C) Conceptual illustration of using a photodegradable hydrogel to create vascular structures within a cell‐encapsulating hydrogel directly at the site of application. Reproduced with permission [283]. Copyright 2017, Royal Society of Chemistry (D) Chemical structure and (E) schematic of sequential cell patterning in a dual‐functional hydrogel system. Reproduced with permission [281]. Copyright 2025, Royal Society of Chemistry. (F) Schematic illustration of hydrogel assemblies dynamically reconfigured by a photo and thermo‐controlled metallopolymer adhesive to achieve multiple customized functions. Reproduced with permission [282]. Copyright 2024, Nature Publishing Group.

### 6.3 Nanomaterials With Dynamic Covalent Chemistry

Dynamic covalent interactions have been widely explored in nanomaterials for biomedical applications, with cancer therapeutics representing a major area of application. More recently, these dynamic chemistries have also been investigated in the context of regenerative medicine, where their reversibility and adaptability can provide additional opportunities to control material–biological interactions. In this context, functionalization with biomimetic molecules can further enhance the biological compatibility and functionality of dynamic nanomaterials [284, 285, 286].

Nanoparticles (NPs) have revolutionized the field of regenerative medicine, offering unique advantages such as high surface area, tunable size, and the ability to deliver therapeutic agents precisely to target tissues [287, 288]. Incorporating DCvC into nanoparticles adds a new layer of functionality by enabling programmable and chemically addressable assembly pathways, providing an important conceptual basis for multiresponsive nanosystems. Leng and coworkers designed hyaluronic acid (HA)–functionalized silica nanoparticles cross‐linked through dynamic phenylboronate ester to achieve dual pH/ROS responsiveness (Figure 16A). The HA shell not only enhanced colloidal stability but also enabled tumor targeting via CD44 receptor binding. These nanohybrids were stable under physiological conditions, however, in acidic and oxidative tumor environments, cleavage of the linkers promoted sustained doxorubicin release. In vitro studies further demonstrated improved cellular uptake and higher cytotoxicity compared to free DOX, underscoring their promise as targeted anticancer carriers [289]. In another study boronate‐ester cross‐linked polymer nanoparticles incorporating dynamic covalent B─O bonds with reversible B←N coordination showed architecture stabilization in circulation while enabling dual responsiveness to acidic pH and intracellular biomolecules/diols. A significant increase in binding affinity (higher *K*_a_) and a decrease in Δ*G* (DFT calculations) confirm that B←N coordination makes boronate ester formation more favorable than B─O systems. Overall, B←N coordination enhances both the kinetics and thermodynamics of boronate ester formation. The dual stimuli also promote lysosomal escape, leading to efficient cytoplasmic delivery of payloads. In vitro, these nanoparticles achieved higher drug loading, controlled release, and stronger anticancer activity compared to free drug (Figure 16B) [290, 291].

**FIGURE 16:**
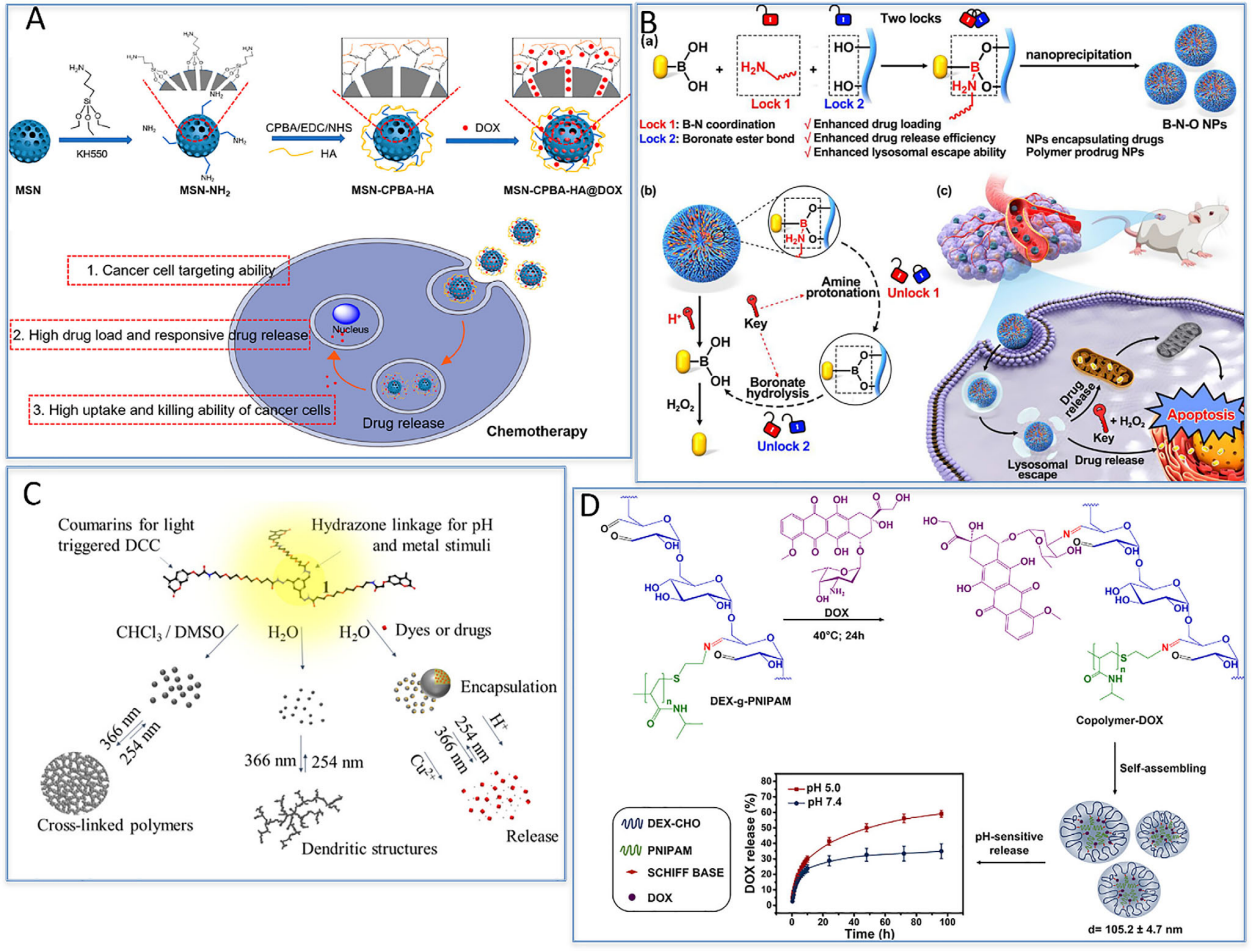
(A) Schematic illustration of the dynamic‐covalent mesoporous silica nanohybrids for precise drug delivery. Copyright 2024, American Chemical Society [289]. (B) Schematic overview of the two‐lock strategy for preparing polymer nanoparticles with enhanced stability, drug loading, responsive release, and lysosomal escape in chemotherapy. Reproduced and adapted with permission [290]. Copyright 2024, American Chemical Society. (C) Dynamic assembly and disassembly of multi‐stimuli‐responsive supramolecular polymers via combined noncovalent and dynamic covalent interactions. Reproduced with permission [57]. Copyright 2020, American Chemical Society. (D) Dual stimuli‐responsive doxorubicin‐loaded dextran‐graft‐PNIPAM nanoparticles for selective drug release under acidic condition in tumor environments. Reproduced with permission [292]. Copyright 2021, Elsevier B.V.

Schiff's base‐engineered nanoparticles can be further tailored by incorporating metal ion coordination and coumarin‐based photodimerization. Hatai and coworkers developed a dynamic polymeric nanoparticle on hydrazone and coumarin functionalized polymer whose self‐assembly showed responsiveness to light, pH, and metal ion coordination. They incorporated noncovalent and dynamic covalent chemistry in their chemical design to generate tri‐substituted benzene derivative. They demonstrated that polymer assemblies can reversibly switch between nanoparticles and networks under light, while acidic conditions cleave hydrazone bonds to disrupt structures, and Cu^2^^+^ ions promote further nanoaggregate disassembly which were translated for drug delivery application. Their polymer showed rare three stimuli responsiveness, opening avenues for multiresponsive polymer developments (Figure 16C) [57]. Likewise, Dextran‐graft‐poly(N‐isopropylacrylamide) copolymers were synthesized via Schiff's base chemistry, and DOX was conjugated through imine linkages to form a prodrug nanoparticle system. The resulting nanoparticles were dual‐responsive: temperature due to PNIPAM and pH due to imine/Schiff base bonds. Drug release was significantly higher at pH 5.0 (∼59%) versus physiological pH (∼35%). In vitro, the DOX‐loaded nanoparticles show good cytotoxicity against cancer cells HCT‐116 and lower toxicity in nontumor cells, with evidence of cellular uptake via endocytosis (Figure 16D) [292]. Another design employs a disulfide‐containing polymer conjugate of branched polyethylenimine (BPEI‐SS‐Pt) that is crosslinked via Schiff base linkages with dialdehyde‐modified PEG to form a nanoparticle. In the acidic tumor microenvironment the Schiff base bonds are cleaved, shedding the PEG shell while in reductive intracellular environments, the disulfide linkages are also cleaved, leading to drug release in a sequential pH‐then redox triggered manner. In vivo experiments showed that these nanoparticles exhibit superior antitumor efficacy compared to free cisplatin, with reduced side effects. The system thus combines dual responses to enhance specificity, and more controlled release [293]. This adaptability makes dynamic covalent nanoparticles ideal candidates for regenerative scaffolds, especially in tissues that undergo constant mechanical or biochemical changes.

To enhance the integration of dynamic nanoparticles with biological systems, functionalization with biomimetic molecules is a critical step. Biomimetic functionalization not only improves the biocompatibility of the nanoparticles but also provides specific cues to guide cellular responses, such as adhesion, differentiation, or proliferation. One effective strategy involves conjugating peptides or proteins that mimic the natural ECM to the surface of dynamic nanoparticles [294, 295, 296, 297]. For instance, the RGD peptide, which binds integrin receptors on cell surfaces, has been widely used to promote cell adhesion and signaling [298, 299]. By coupling RGD peptides to dynamic nanoparticles through reversible covalent bonds, researchers have created systems that dynamically regulate cell adhesion and detachment in response to environmental cues.

Antimicrobial nanoparticles based on multiple dynamic covalent interactions—specifically imine formation and boronic acid–catechol (polyphenol) chemistry—were developed by Yuanfeng Li, Yin‐Zi Piao, and Hua Chen (Figure 17A,B) [300]. These materials notably alleviated the intrinsic cytotoxicity associated with multiamine‐containing antibiotics and effectively prevented the oxidation of the polyphenols. Another anibacterial nanoparticle system was formulated on Omiganan (antibacterial peptide) and dexamethasone (hydrophilic and hydrophobic respectively, to form amphiphilic system) via hydrazone bond, which then coated with HA for target specificity and enzyme responsiveness making it sensitive to pH and enzyme: dual responsive. Omi‐hyd‐Dex@HA nanoparticles showed antimicrobial activity against bacteria and fungi, while effectively suppressing inflammation and neutralizing LPS with excellent biocompatibility. Guided by HA/ICAM‐1 targeting, they accumulated at infected sites and markedly reduced sepsis mortality in mice highlighting their promise as a self‐delivering nano‐therapeutic for frontline sepsis treatment (Figure 17C) [301].

**FIGURE 17:**
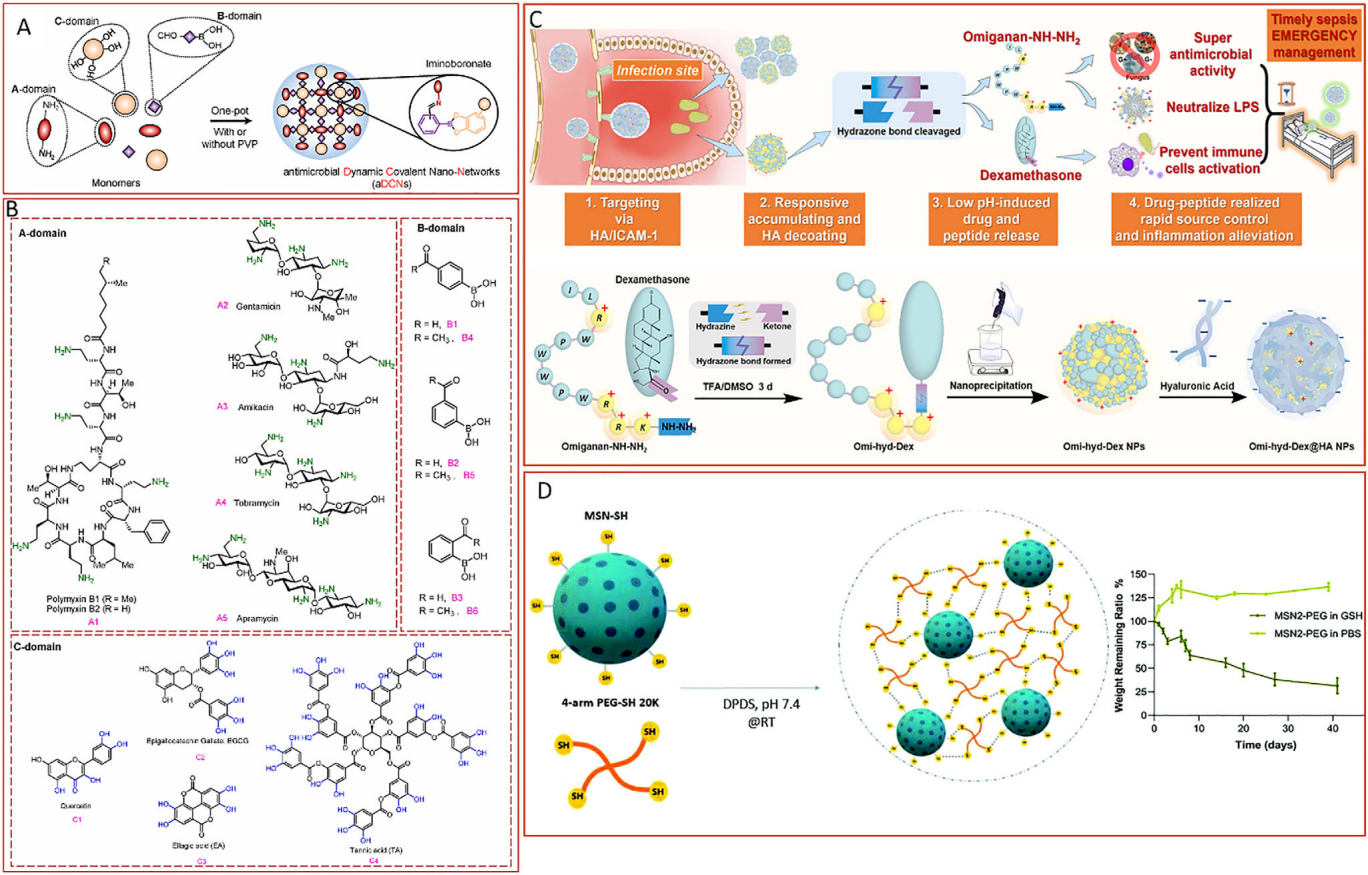
(A) and (B) Chemical structures of multiarm amines (A domains), carbonyl‐containing aryl boronic acids (B domains), and selected polyphenols/polycatechols (C domain) used in the synthesis of antimicrobial dynamic covalent nano‐networks. Reproduced with permission [300]. Copyright 2023, Elsevier B.V. (C) Flow chart of the polymer nanoparticle application, mechanism and synthesis. Reproduced and adapted with permission [301]. Copyright 2023, Elsevier B.V. (D) Nanoparticle based hydrogel synthesis via dynamic covalent interactions of MSN‐SH and 4‐arm‐PEG‐SH through thiol–disulfide exchange reactions and degradation profile with GSH. Reproduced and adapted with permission [302]. Copyright 2021, Royal Society of Chemistry.

There is growing interest in hybrid nanomaterials that combine nanoparticles with hydrogels for a variety of biomedical applications, including 3D printing and wound healing. Van Rijt and team developed mesoporous nanoparticles with thiol functionalization as reversible crosslinker in PEG hydrogel. This impart rapid self healing in the hydrogel due to reversible disulphide bonds and redox responsive degradation in presence of GSH. The nanocomposite also showed 25 fold increase in mechanical strength while renaining stable under physilogical conditions (Figure 17D) [302].

In a study, amphiphilic copolymer of hyaluronic acid (HA) which was conjugated to stearic acid through hydrazone and disulfide linkages, producing self‐assembled nanomicelles that are pH‐ and redox‐dual responsive were designed and synthesized. The hydrazone bonds cleave under acidic conditions (tumor endosomes), while disulfide bonds are reduced intracellularly by GSH, together enabling staged cargo release. HA acts as a biomolecular functionalization that both improves aqueous stability/bioavailability and provides active CD44‐mediated targeting [303].

## 7 Outlook and Perspective

The history of biomaterials for regenerative medicine can be interpreted as a progression through successive generations of increasing complexity [8, 304]. Each generation addressed key limitations of the previous one, while simultaneously revealing new requirements imposed by the complexity of biological systems. It has progressed from simple structural scaffolds to bioactive platforms that support tissue regeneration and therapeutic delivery [305, 306, 307]. As these functions became more sophisticated, it became clear that the next generation of materials must not only provide biological support but also dynamically adapt to changing physiological environments by integrating multiple environmental cues [87, 308, 309]. This further degree of complexity is certainly necessary in order to understand native ECM: to understand it, you need to recreate it [310, 311]. This is only possible through modern tools such as computer simulations and AI, 3D printing, and microscopy, which push the boundaries of what we can understand, because they allow us not only to observe things, but also to reproduce them ourselves [312, 313, 314, 315].

Early hydrogel‐like materials defined the modern definition of the term. Ivalon, a formaldehyde‐crosslinked poly(vinyl alcohol) sponge from the late 1940s, and rigid PMMA contact lenses represent an earlier generation, though both suffered from limited hydrophilicity or biocompatibility [316, 317]. The term “hydrogel” is generally dated back to Wichterle and Lim's 1954 report on crosslinked pHEMA, whose hydrated, biocompatible network led to FDA‐approved soft contact lenses by 1971 and later applications in controlled drug delivery [316, 318]. A related milestone came in 1967, when Updike and Hicks entrapped glucose oxidase in a polyacrylamide hydrogel to create an early enzyme‐based biosensor [316, 319]. Building on this foundation, a subsequent generation of hydrogel biomaterials for regenerative applications turned to more defined static covalent architectures, including thiol–Michael PEG networks, carbodiimide‐crosslinked collagen and alginate, methacryloyl‐gelatin systems, and fibrin formed via the coagulation cascade [320, 321, 322]. Despite differing chemistries, these systems shared a common design logic: The network was formed once, under controlled conditions, and thereafter remained structurally stable or predictable [323, 324]. These materials performed their intended roles as passive structural supports environments that cells could colonize, migrate through, and remodel enzymatically, but that could not themselves adapt, signal back, or change in response to what the cells were doing. The limitation was not incidental to the design; it was inherent to the chemistry a static covalent bond, once formed, offers few mechanisms for adaptation [325, 326].

The introduction of dynamic covalent chemistry into biomaterial design represented a genuine conceptual departure from this paradigm [108, 247]. These single‐stimulus responsive systems opened important new possibilities: self‐healing wound dressings, environment‐triggered drug release platforms, and injectable scaffolds that gel in situ and established dynamic covalent chemistry as a credible design language for soft biomaterials [327]. This single degree of freedom was not a shortcoming in itself, many applications need a material to do exactly one thing, reliably. Its limitation only becomes apparent against the benchmark of living tissue, which continuously remodels in response to multiple, simultaneous cues rather than a single trigger [328, 329].

This distinction matters because biological systems rarely rely on a single cue: cells proliferate, differentiate, and are maintained optimally under distinct, often sequential microenvironmental conditions, a complex no “single‐stimulus material” can mimic. For example, it is very complex to create a material that is equally suitable for cell proliferation and differentiation [330, 331, 332, 333]. The same challenge arises in translational contexts, a formulation optimized for cold‐chain storage, for instance, may be entirely unsuited to subsequent targeted delivery, yet only one material system is available to satisfy both demands across its lifecycle. However, it is precisely here that the advances in (bio)orthogonal chemistry over the last 20 years deserve to be highlighted [79]. We can add stimuli responsiveness that the cells can produce (endogenous), but also stimuli that we can control (exogenous) [197, 334, 335]. This means that, compared to nature, we have yet another degree of control: we can create or break bonds, release molecules, and so on, on demand, via stimuli such as light, ultrasound, or exogenous functional molecules that we add. It is this need to perform multiple, context‐dependent functions within a single system, not merely to respond to change, but to respond appropriately to different changes, that positions multistimuli responsive dynamic covalent materials as a categorical necessity rather than an incremental refinement.

What multistimuli responsive systems offer, beyond simply broader sensitivity, is the capacity for information integration [336]. A material incorporating two (or more) orthogonal dynamic covalent chemistries, for instance, a disulfide network that reacts under reducing conditions combined with a Schiff‐base crosslink that hydrolyzes at mildly acidic pH, does not merely respond to two things; it responds differently depending on which combination of signals it receives [253]. We can add endogenous stimuli, that the cells can produce, but also exogenous stimuli that we can control. It can, in principle, implement rudimentary chemical logic: stiffening under mechanical load while simultaneously releasing a sequestered growth factor upon enzymatic cleavage, or degrading site‐specifically only where both elevated reactive oxygen species and lowered pH coincide, a molecular AND gate that no single‐responsive system can instantiate [337, 338, 339, 340]. This represents a qualitative shift in what a synthetic biomaterial can be asked to do, moving from a passive responder to a material that actively negotiates with its cellular environment, integrating contextual information and producing outputs calibrated to the composite state of the tissue rather than to any single input. Ultimately, such materials could move beyond sensing and adaptation to become active therapeutic systems that locally generate, release, or activate therapeutics in response to tissue‐specific cues, effectively functioning as responsive therapeutic depots.

This added functionality is accompanied by a marked increase in mechanistic complexity, as the coupled exchange kinetics and thermodynamics of multiple, simultaneously responsive bonds remain poorly captured by current analytical and theoretical frameworks [341].

When a network incorporates only a single dynamic bond, its mechanical and chemical behavior can, at least in principle, be characterized within emerging and recently established framework. Double‐dynamic systems need to be understood in more depth. Now we have emerging tools to understand them better. Stress‐relaxation rheology yields a characteristic bond or crosslink lifetime; spectroscopic titration quantifies the equilibrium constant; network models derived from classical rubber elasticity or transient network theory can, with appropriate modifications, start to describe the resulting viscoelastic properties [191, 339, 342, 343, 344]. None of this extends straightforwardly to multibond systems. When two or more dynamic chemistries operate simultaneously within the same network, their exchange kinetics are not independent: network topology couples their relaxation dynamics such that the apparent rate of one bond type is constrained by the conformational and mechanical state imposed by the other [109, 345]. Even competitive exchange within a single bond type is only starting to be mechanistically understood, while the orthogonality, or interference, between distinct dynamic chemistries in dual‐ or multi‐dynamic systems remains largely unexplored [104, 346].

The two populations of crosslinks mutually influence each other's effective kinetics in ways that emerge from network architecture rather than from the intrinsic chemistry of either bond alone, an interplay that remains an area for exploration.

Thermodynamically the equilibrium crosslink distribution in a multibond network, and therefore its mechanical state at any given moment, is determined by the coupled equilibria of all dynamic species simultaneously, including competitive reactions with solvent, biological nucleophiles such as amines or thiols present, for example in serum, and adventitious oxidants [347]. This coupling introduces a form of path‐dependence: the same material, exposed to the same final set of conditions by different routes, may reach different metastable states depending on the order and timing of stimulus application. This is not a design flaw but a reflection of the genuine complexity that arises when multiple reversible chemistries coexist, and it underscores why empirical optimization, varying composition and measuring bulk properties, becomes increasingly inadequate as a design strategy when the number of coupled equilibria grows. Rheology captures the bulk viscoelastic consequences of bond exchange but cannot attribute changes in storage or loss modulus to specific bond populations within a multicomponent network, without solid theory development or molecular model studies [348, 349, 350]. Solid‐state NMR exchange spectroscopy, bond‐specific infrared or Raman signatures, and small‐molecule fluorescent reporters for individual dynamic species each illuminate a partial view, but integrating these orthogonal datasets into a mechanistically coherent picture of how a multiple responsive network behaves under physiologically realistic conditions remains technically demanding and rarely attempted in the literature [351, 352, 353, 354, 355]. Developing theoretical frameworks capable of predicting emergent network behavior from the individual thermodynamic and kinetic parameters of constituent bond types is one of the most important unresolved methodological challenges facing the field, and progress here will be essential for moving from materials discovered by exploration to materials designed by intention [356, 357, 358, 359].

Looking further ahead, the most consequential implication of multistimuli responsive dynamic covalent materials for regenerative medicine is not that they are more responsive than their predecessors, but that they are, for the first time, architecturally capable of behaving as instructive rather than merely reactive environments by drawing on two different classes of stimuli (endogenous and exogenous), and therefore even possess a capability that nature itself does not have [335]. Native extracellular matrices do not simply respond to cells; they encode and transmit information presenting adhesion ligands, sequestering growth factors, and remodeling their mechanical landscape in a manner that is continuously updated by cellular activity and that in turn directs cell fate decisions across proliferation, differentiation, and migration [192, 352]. A synthetic material capable of integrating multiple simultaneous inputs and producing coordinated, context‐dependent outputs begins to approximate this function in a way that no single‐stimulus system can. Whether such materials will ultimately be capable of the richness and adaptability of biological matrices remains an open question, but the chemical and conceptual foundations for pursuing it are now in place. However, we need to be careful not to over‐engineer and make sure to use materials that have pathways toward scalability for translation to industry and the clinic.

We need to ask ourselves what problems we want to solve. Do we need another stimulus? Do we need the ability to sense? And if the approach is that we want to understand native ECM in order to ultimately rebuild it, then our answer is clearly yes. The ambition for the next decade is to build the analytical, theoretical, and synthetic scaffolding that would allow these materials to be not just demonstrated, but genuinely understood and rationally designed, and in doing so, to bring the third generation of biomaterials a decisive step closer to the clinic.

## Author Contributions

**Saurabh Joshi**: writing – original draft, conceptualization, validation, visualization. **Matthew B. Baker**: supervision, conceptualization, writing – original draft, writing – review and editing, visualization, validation, project administration. **Dominik Schauenburg**: conceptualization, writing – review and editing, writing – original draft, supervision, validation, visualization, project administration.

## Conflicts of Interest

MBB is co‐founder, shareholder, and CSO of MosaMatrix B.V. and hold patents based on dynamic covalent biomaterials.

## Data Availability

Research data are not shared.
